# Chronic UCN2 treatment desensitizes CRHR2 and improves insulin sensitivity

**DOI:** 10.1038/s41467-023-39597-w

**Published:** 2023-07-04

**Authors:** Stephen E. Flaherty, Olivier Bezy, Wei Zheng, Dong Yan, Xiangping Li, Srinath Jagarlapudi, Bina Albuquerque, Ryan M. Esquejo, Matthew Peloquin, Meriem Semache, Arturo Mancini, Liya Kang, Doreen Drujan, Susanne B. Breitkopf, John D. Griffin, Pierre M. Jean Beltran, Liang Xue, John Stansfield, Evanthia Pashos, Quazi Shakey, Christian Pehmøller, Mara Monetti, Morris J. Birnbaum, Jean-Philippe Fortin, Zhidan Wu

**Affiliations:** 1 Internal Medicine Research Unit, Pfizer Inc., 1 Portland Street, Cambridge, MA USA; 2 Domain Therapeutics North America, Montréal, QC Canada; 3 Machine Learning and Computational Sciences, Pfizer Inc., 1 Portland Street, Cambridge, MA USA; 4 Biostatistics, Early Clinical Development, Pfizer Inc., 1 Portland Street, Cambridge, MA USA; 5 Biomedicine design, Pfizer Inc., 1 Portland Street, Cambridge, MA USA

**Keywords:** Metabolism, Metabolic diseases

## Abstract

Urocortin 2 (UCN2) acts as a ligand for the G protein-coupled receptor corticotropin-releasing hormone receptor 2 (CRHR2). UCN2 has been reported to improve or worsen insulin sensitivity and glucose tolerance in vivo. Here we show that acute dosing of UCN2 induces systemic insulin resistance in male mice and skeletal muscle. Inversely, chronic elevation of UCN2 by injection with adenovirus encoding UCN2 resolves metabolic complications, improving glucose tolerance. CRHR2 recruits Gs in response to low concentrations of UCN2, as well as Gi and β-Arrestin at high concentrations of UCN2. Pre-treating cells and skeletal muscle ex vivo with UCN2 leads to internalization of CRHR2, dampened ligand-dependent increases in cAMP, and blunted reductions in insulin signaling. These results provide mechanistic insights into how UCN2 regulates insulin sensitivity and glucose metabolism in skeletal muscle and in vivo. Importantly, a working model was derived from these results that unifies the contradictory metabolic effects of UCN2.

## Abstract (web summary)

UCN2 acts as a ligand for the GPCR CRHR2 and there have been conflicting reports on whether UCN2 treatment improves or worsens glucose tolerance. Here, the authors show that acute UCN2 recruits Gs and decreases glucose uptake, while chronic treatment desensitizes CRHR2 and improves glucose uptake.

## Introduction

Urocortins (UCNs) are members of the corticotropin releasing hormone (CRH) family, which includes UCN1, UCN2, and UCN3. All three are established neuroendocrine signaling peptides regulating physiological responses to stress via the hypothalamic-pituitary-adrenal axis (HPA)^1,2^. More recently, these peptides have been found to have diverse roles outside of the central nervous system, including mediating apoptosis, regulating inflammation, improving heart function, and inhibiting lipolysis^3–7^. UCNs carry out their signaling through binding to the G protein-coupled receptors (GPCRs), Corticotropin Releasing Hormone Receptor 1 (CRHR1) and 2 (CRHR2)^8^. UCN1 binds to both CRHR1 and CRHR2, while UCN2 and UCN3 bind exclusively to CRHR2^9,10^. Unlike CRHR1, CRHR2 is expressed in the central nervous system (CNS) as well as in several peripheral tissues^11^.

UCN2 and UCN3 have been implicated in various aspects of energy balance and metabolism. Intracerebroventricular injection of UCN2 and UCN3 suppresses motor activity and food intake^12^. UCN3-overexpressing mice are protected from diet-induced obesity (DIO) and have enhanced insulin signaling and glucose sensitivity^13,14^. Studies on the effects of UCN2 on insulin sensitivity, however, have been contradicting. Germline CRHR2 KO mice have improved glucose tolerance^15^. Germline UCN2 KO animals have improved glucose tolerance that is worsened following UCN2 re-administration^16^. Treatment of animals with CRHR2 antagonists improves glucose tolerance^16^ and humans in the upper quartile range of circulating UCN2 have increased risk of insulin resistance^17^. These findings indicate a role for UCN2 as an insulin desensitizer. In contrast, animals overexpressing UCN2 while on high-fat diet and animals receiving a daily subcutaneous dose of PEGylated UCN2 have improved glucose tolerance, pointing to UCN2 as an insulin sensitizer^18,19^.

Type-II GPCRs like CRHR2 typically transduce signal through activation of protein Gs, leading to increased intracellular cAMP concentrations and phosphorylation of downstream target proteins^20^. Persistent GPCR stimulation can be deleterious, however, resulting in cellular toxicity or uncontrolled growth, and thus nature has developed mechanisms to regulate GPCR activation through homologous receptor desensitization. To initiate desensitization, G protein-coupled receptor kinases (GRKs) are recruited to the cell membrane. GRKs phosphorylate the intracellular domain of the ligand-bound receptor attracting β-Arrestins to compete with Gs for binding, sterically hindering downstream transduction of the signal. In cases of chronically sustained Type II GPCR activation, β-Arrestins can induce the internalization of the GPCR through clathrin-mediated endocytosis, insulating the receptor from extracellular signals. The regulation of Type II GPCRs through these diverse mechanisms can result in different downstream biological effects. Some previous studies have shown Gs-alternative signaling and potential internalization of CRHR2^21–24^.

In this study we examined the effects of CRHR2 signal transduction on systemic and local insulin sensitivity. We monitored glucose homeostasis in response to CRHR2 activation by UCN2 and, strikingly, found that acute treatment of UCN2 induces glucose intolerance in mice, while chronic treatment of UCN2 improves glucose tolerance. This phenomenon persisted in ex vivo isolated peripheral tissues and in vitro cell culture systems. We observed that each human CRHR2 isoform effectively recruits Gs in response to low UCN2 concentrations. At high concentrations of UCN2, CRHR2 also engaged Gi/o and β-Arrestins. The engagement of inhibitory proteins and β-Arrestins, known to promote receptor desensitization, potentially serves as an explanation for the divergent effects of UCN2 on insulin signaling when treated either acutely or chronically. Overall, this study has important implications for the potential use of CRHR2 antagonists for treatment of insulin resistance and offers insights into the molecular relationship between insulin’s effects and GPCR signal transduction.

## Results

### Increased circulating UCN2 and UCN3 in models of insulin resistance

**Fig. 1:**
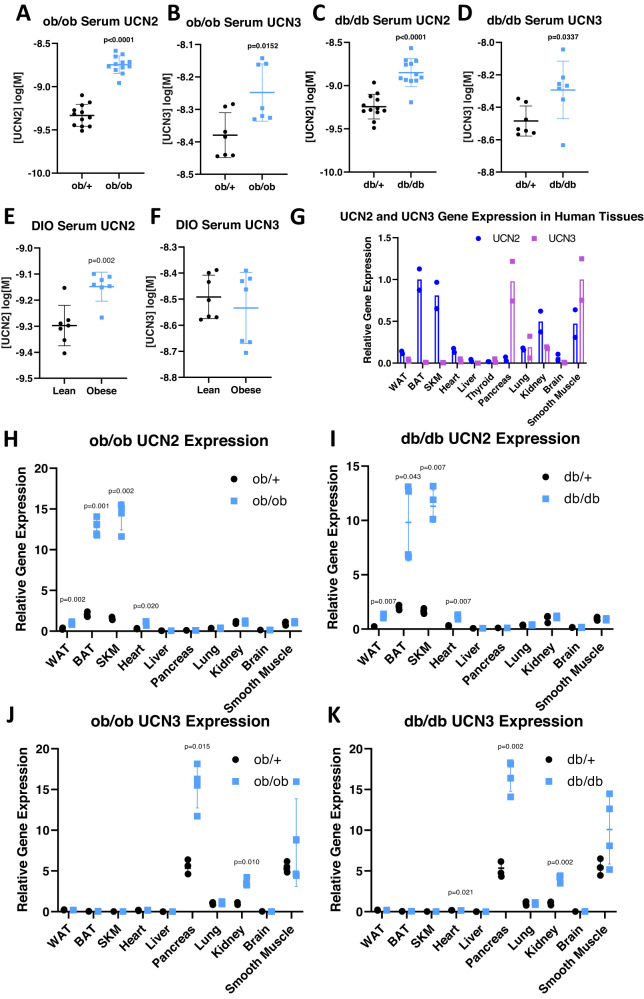
UCN2 gene expression in metabolic tissues and circulating UCN2 levels are associated with insulin resistance. **A** Serum UCN2 levels in ob/ob animals. *n* = 12. Significance was assessed by Welch’s two-way two sample *t*-test. **B** Serum UCN3 levels in ob/ob animals. *n* = 7. Significance was assessed by Welch’s two-way two sample *t*-test. **C** Serum UCN2 levels in db/db animals. *n* = 12. Significance was assessed by Welch’s two-way two sample *t*-test. **D** Serum UCN3 levels in db/db animals. *n* = 7. Significance was assessed by Welch’s two-way two sample *t*-test. **E** Serum UCN2 levels in DIO animals. *n* = 7. Significance was assessed by Welch’s two-way two sample *t*-test. **F** Serum UCN3 levels in DIO animals. *n* = 7. Significance was assessed by Welch’s two-way two sample *t*-test. **G** Relative gene expression of *ucn2* and *ucn3* in human tissues. *n* = 2 biologically independent samples. **H** Relative gene expression of *ucn2* in tissues from ob/ob animals. *n* = 4. Significance was assessed by Welch’s two-way two sample *t*-test for each tissue. **I** Relative gene expression of *ucn2* in tissues from db/db animals. *n* = 4. Significance was assessed by Welch’s two-way two sample *t*-test for each tissue. **J** Relative gene expression of *ucn3* in tissues from ob/ob animals. *n* = 4. Significance was assessed by Welch’s two-way two sample *t*-test for each tissue. **K** Relative gene expression of *ucn3* in tissues from db/db animals. *n* = 4. Significance was assessed by Welch’s two-way two sample t-test for each tissue. All Data are presented as mean values +/− SD. Source data are provided as a Source Data file.

Given the published effects that UCN2 and UCN3 have on insulin signaling, we began by investigating circulating UCN2 and UCN3 levels in established genetic models of insulin resistance. Leptin-deficient (*ob/ob*) animals have elevated levels of circulating UCN2 and UCN3 (Fig. 1A, B). Leptin receptor-deficient (*db/db*) animals, likewise, have elevated UCN2 and UCN3 serum levels (Fig. 1C, D). UCN2 serum levels were also elevated in diet-induced obese (DIO) WT mice fed a high-fat diet for 20 weeks, while UCN3 levels remained unchanged (Fig. 1E, F). Gene expression analysis in human tissue indicated that *ucn2* is mostly produced in the brown fat, skeletal muscle, kidney, and smooth muscle, while *ucn3* is produced mostly in the pancreas and smooth muscle (Fig. 1G).

To determine the source of the UCNs in these insulin-resistant models, gene expression patterns were determined for *ob/ob* and *db/db* tissues. *Ucn2* expression was increased in the white adipose tissue, brown adipose tissue, heart, and skeletal muscle (Fig. 1H, I). *Ucn3* expression, on the other hand, was significantly increased in the pancreas and kidney in both insulin-resistant models (Fig. 1J, K). This indicates that the majority of the increased circulating UCN2 in obese animals is contributed by skeletal muscle and BAT.

### Acute UCN2 treatment induces insulin resistance

**Fig. 2:**
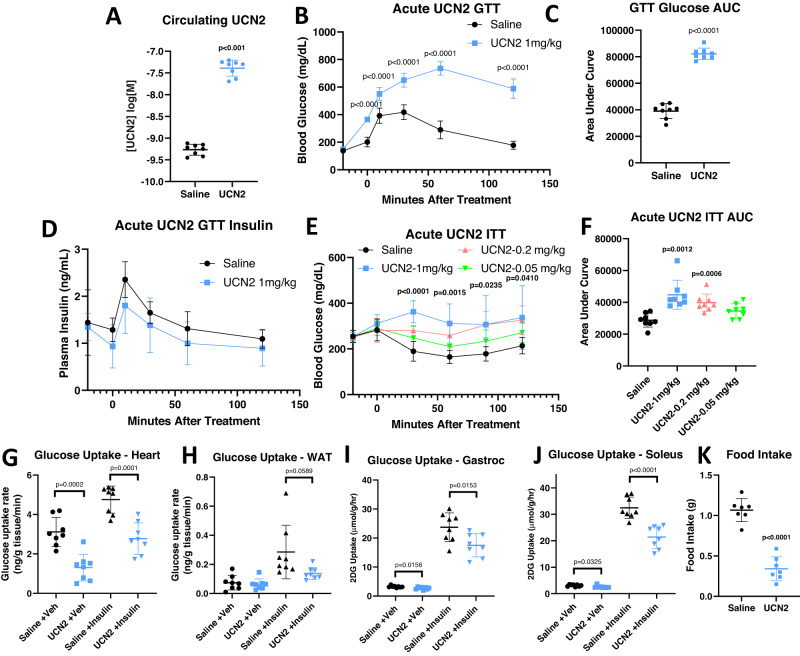
Acute UCN2 treatment impairs glucose uptake. **A** Serum UCN2 levels in WT animals 20 min after saline or 1 mg/kg UCN2 dose. *n* = 8. Significance was assessed by Welch’s two-way two sample *t*-test. **B** Blood glucose levels during an oral glucose tolerance test (oGTT) in mice pre-treated with UCN2. *n* = 7. Significance was assessed by Welch’s two sample *t*-test. **C** Area under the curve (AUC) of IPGTT shown in (**B**). *n* = 7. Significance was assessed by Welch’s two-way two sample *t*-test. **D** Plasma insulin levels during an IPGTT in mice pre-treated with UCN2. *n* = 7. **E** Blood glucose levels during an insulin tolerance test (ITT) in mice pre-treated with varying doses of UCN2. *n* = 8. Significance was assessed by one-way ANOVA and Tukey HSD. **F** Area under the curve (AUC) of ITT shown in (**E**). *n* = 8 biologically independent animals. Significance was assessed by one-way ANOVA and Tukey HSD. **G** FDG uptake by the heart in insulin-treated or untreated mice, following pre-treatment of UCN2 or saline. *n* = 8. Significance was assessed by one-way ANOVA and Tukey HSD. **H** FDG uptake by the subcutaneous white adipose tissue in insulin-treated or untreated mice, following pre-treatment of UCN2 or saline. *n* = 8. Significance was assessed by one-way ANOVA and Tukey HSD. **I** [^3^H]2-Deoxyglucose uptake by the gastrocnemius muscle in insulin-treated or untreated mice, following pre-treatment of UCN2 or saline. *n* = 8. Significance was assessed by one-way ANOVA and Tukey HSD. **J** [^3^H]2-Deoxyglucose uptake by the soleus muscle in insulin-treated or untreated mice, following pre-treatment of UCN2 or saline. *n* = 8. Significance was assessed by one-way ANOVA and Tukey HSD. **K** Food intake of mice treated with a single dose of UCN2 or saline. *n* = 7. Significance was assessed by Welch’s two-way two sample t-test. All data are presented as mean values +/− SD. Source data are provided as a Source Data file.

To validate the direct effects of UCN2 on glucose metabolism, mice were treated acutely with a single intraperitoneal (IP) injection of 1 mg/kg human UCN2 recombinant protein. Twenty minutes after injection, circulating concentrations of UCN2 reached an average of 40 nM (−7.4 log[M]) (Fig. 2A). Oral glucose tolerance tests (oGTTs) performed twenty minutes after UCN2 injection showed that mice treated with UCN2 displayed glucose intolerance, with highly elevated glucose levels two hours after the glucose bolus (Fig. 2B, C). No significant difference in plasma insulin levels between the vehicle and UCN2-treated animals during the oGTT was detected (Fig. 2D). To determine whether the decreased glucose tolerance was primarily due to insulin resistance or a decrease in insulin secretion, insulin tolerance tests (ITTs) were performed. ITTs demonstrated that increasing doses of UCN2 gave rise to decreasing rates of glucose uptake in response to insulin injection (Fig. 2E, F), confirming that acute UCN2 causes insulin resistance in vivo. UCN2 treatment decreased insulin-stimulated glucose uptake in the heart, white adipose tissue (WAT), and SKM in vivo (Fig. 2G–I). Consistent with previous literature, a decrease in food intake was also observed following UCN2 dosing (Fig. 2K)^12,25^.

To determine whether the effects of UCN2 on glucose metabolism were driven through its action in the CNS or periphery, animals were pre-treated with Antisauvagine-30 (ASG30), a CRHR2-selective antagonist that does not cross the blood brain barrier^26,27^. ASG30 significantly blunted UCN2’s effects on blood glucose (Fig. S1A). During oGTT, ASG30 pre-treatment improved glucose tolerance in UCN2-treated animals (Fig. S1A). ASG30 pre-treatment also restored insulin-mediated glucose uptake in the soleus muscle in vivo (Fig. S1B). These experiments demonstrate that UCN2’s effects on glucose uptake are primarily driven by the peripheral action of CRHR2.

### Chronic UCN2 treatment improves glucose tolerance and insulin sensitivity

**Fig. 3:**
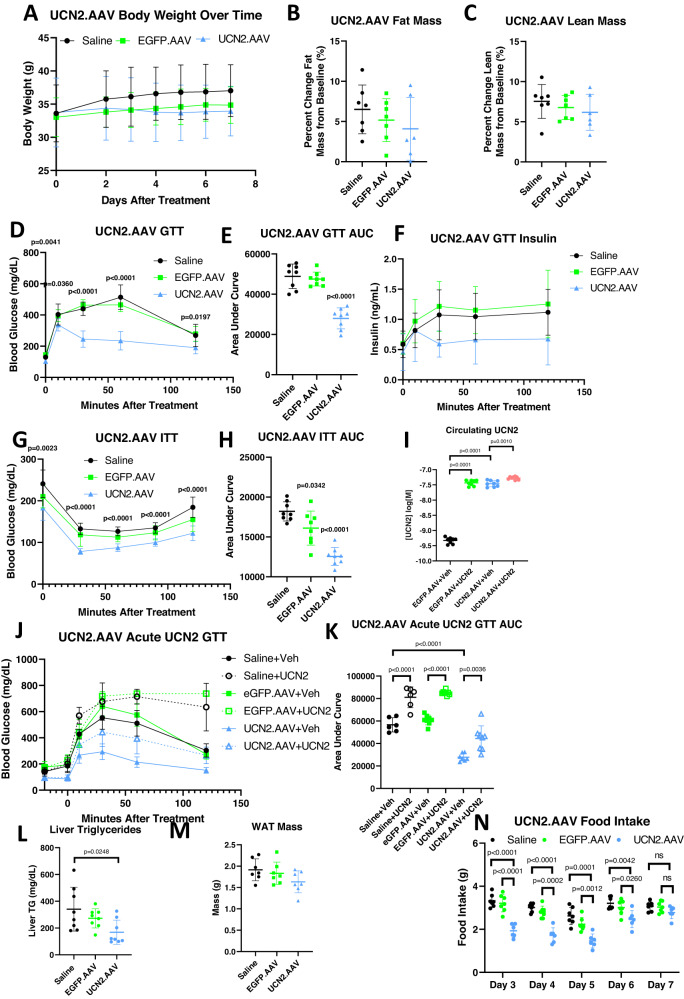
Chronic UCN2 treatment improves glucose sensitivity. **A** Body weights of Saline, EGFP.AAV, and UCN2.AAV animals after initiation of treatment. *n* = 7. **B** Percent change in fat mass of Saline, EGFP.AAV, and UCN2.AAV animals 7 days after initial treatment. *n* = 7. **C** Percent change in lean mass of Saline, EGFP.AAV, and UCN2.AAV animals 7 days after initial treatment. *n* = 7. **D** Blood glucose levels during an intraperitoneal glucose tolerance test (IPGTT) in Saline, EGFP.AAV, and UCN2.AAV animals 7 days after initial treatment. *n* = 7. Significance was assessed by one-way ANOVA and Tukey HSD. **E** Area under the curve (AUC) of IPGTT shown in (**D**). *n* = 7. Significance was assessed by one-way ANOVA and Tukey HSD. **F** Plasma insulin levels during an IPGTT in mice pre-treated with UCN2. *n* = 7. **G** Blood glucose levels during an insulin tolerance test (ITT) in Saline, EGFP.AAV, and UCN2.AAV animals 7 days after initial treatment. *n* = 8. Significance was assessed by one-way ANOVA and Tukey HSD. **H** Area under the curve (AUC) of ITT shown in (**G**). *n* = 8. Significance was assessed by one-way ANOVA and Tukey HSD. **I** Circulating UCN2 levels in EGFP.AAV or UCN2.AAV mice treated with UCN2 or saline. *n* = 8. Significance was assessed by two-way pairwise wilcoxon test. **J** Blood glucose levels during an intraperitoneal glucose tolerance test in Saline, EGFP.AAV, or UCN2.AAV animals treated with saline or UCN2. *n* = 8. **K** Area under the curve (AUC) of IPGTT shown in (**J**). *n* = 8. Significance was assessed by one-way ANOVA and Tukey HSD. **L** Liver triglyceride levels of Saline, EGFP.AAV, or UCN2.AAV animals 7 days after initial treatment. *n* = 8. Significance was assessed by one-way ANOVA and Tukey HSD. **M** Epididymal white adipose tissue weights of Saline, EGFP.AAV, or UCN2.AAV animals 7 days after initial treatment. *n* = 8. Significance was assessed by one-way ANOVA and Tukey HSD. **N** Daily food intake levels Saline, EGFP.AAV, or UCN2.AAV animals. *n* = 6. Significance was assessed by one-way ANOVA. All Data are presented as mean values +/− SD. Source data are provided as a Source Data file.

To evaluate the effects of chronic UCN2 treatment on whole body glucose metabolism, an AAV8 virus was utilized to overexpress UCN2 in mouse liver (Fig. S2A). Circulating levels of UCN2 in AAV-treated animals rose significantly two days after treatment and continued to rise for seven days following treatment (Fig. S2B). Overexpressing UCN2 (UCN2.AAV) did not significantly change body weight, fat mass, and lean mass during the course of the seven-day study (Fig. 3A–C). On day seven, a GTT was performed, and surprisingly, we saw the opposite effect of acute UCN2 dosing (Fig. 2). Animals overexpressing UCN2 displayed significantly improved glucose tolerance compared to animals that received a control virus (EGFP.AAV) (Fig. 3D, E). These animals also showed a statistically insignificant trend towards decreased circulating insulin levels, following glucose injection (Fig. 3F). UCN2.AAV animals displayed improved insulin sensitivity during ITT (Fig. 3G, H). Viral injection achieved a circulating concentration of UCN2 comparable to levels observed 20 min following an IP dose of 1 mg/kg of recombinant UCN2 protein (Fig. 3I). Injecting UCN2.AAV animals, acutely, with UCN2 increased circulating levels further and worsened glucose tolerance, again showing that acute UCN2 treatment impairs glucose uptake (Fig. 3I–K). UCN2.AAV liver triglycerides were significantly decreased and epididymal adipose tissue mass trended in the same direction, which could potentially contribute to the improvement in glucose uptake (Fig. 3L, M). Although UCN2.AAV animals initially displayed reductions in food intake, this effect faded over time, such that after seven days following treatment with the virus, there was no difference in food intake between the groups (Fig. 3N), despite circulating levels of UCN2 remained elevated (Fig. 3I).

### UCN2 activates CRHR2 Gs coupling at low concentrations and Gi and β-arrestin coupling at high concentrations

The reversal of UCN2’s effects on glucose uptake and the blunting over time of UCN2’s effects on food intake suggested the possibility of CRHR2 desensitization. Typically, GPCR desensitization is carried out through the recruitment of β-Arrestins or alternative G-proteins to the receptor. To identify whether these proteins are recruited to CRHR2 in response to UCN2, enhanced bystander bioluminescence resonance energy transfer (ebBRET)-based effector membrane translocation assays (EMTA) were carried out in HEK293 cells^28,29^. Cells were co-transfected with plasmids expressing unmodified CRHR2a (alpha isoform) and one of various pathway-specific biosensors. The G protein biosensors (except Gs) consist of three core components: (i) C-terminal *Renilla* luciferase (RlucII)-tagged sub-domains of various G protein effector proteins that selectively interact with active Gα subunits in a G protein family-selective manner_,_ (ii) a plasma membrane (PM) anchored *Renilla* GFP (rGFP), and (iii) an unmodified Gα protein subtype. Upon GPCR (and subsequent G protein) activation, the RlucII-tagged effector proteins translocate to the PM, bringing RlucII in close physical proximity to the PM-anchored rGFP and thus producing an increase in ebBRET. The same translocation principle is used for the β-Arrestin assays, whereby RlucII-tagged β-Arrestins 1 and 2 translocate to the PM following receptor activation. Exceptionally for the Gs biosensor, the Gas protein is directly fused to RlucII^30^. This is due to the lack of a selective soluble downstream effector of Gs that could be used in the same configuration as the other aforedescribed G protein biosensors. Gs activation provokes its dissociation from the PM, resulting in a spatial separation of Gas-tagged RlucII and PM-achored rGFP and a subsequent reduction in ebBRET^31,32^.

**Fig. 4:**
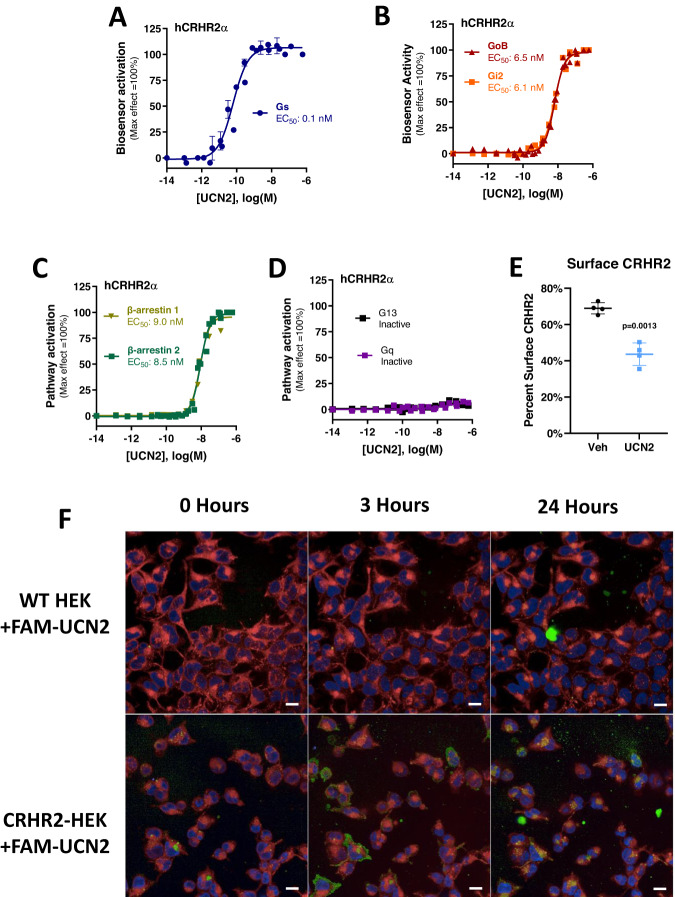
UCN2 treatment recruits Gs, Gi, and β-arrestins. **A** CRHR2a Gs recruitment as a factor of increasing UCN2 dose in HEK293 cells. *n* = 3 independent experiments. **B** CRHR2a Gi and Go recruitment as a factor of increasing UCN2 dose in HEK293 cells. *n* = 3 independent experiments for Gi. *n* = 2 independent experiments for Go. **C** CRHR2a β-Arr1 and β-Arr2 recruitment in response to increasing UCN2 concentrations in HEK293 cells. *n* = 3 independent experiments for β-Arr1. *n* = 2 independent experiments for β-Arr2. **D** CRHR2a Gq, G13 recruitment in response to increasing UCN2 concentrations in HEK293 cells. *n* = 2 independent experiments. **E** Percent CRHR2 on the surface of transiently transfected HEK293 cells treated with Saline or UCN2. *n* = 4 independent experiments. Significance was assessed by Welch’s two-way two sample *t*-test. **F** Fluorescence microscopy images of HEK293 cells transiently transfected with HA-tagged CRHR2a treated with saline or fluorescently labeled UCN2 (FAM-UCN2) over the course of 24 h. Experiments were repeated independently 4 times. Blue = Nuclei, Red = Lysosomes, Green = FAM-UCN2. Scale bars = 10 µm. All data are presented as mean values +/− SD.

Through this process, it was determined that Gs is readily activated by human CRHR2a in response to UCN2 with an EC_50_ of 0.1 nM (−10.0 log[M]) (Fig. 4A). We also observed the recruitment of β-Arrestins 1 and 2 at an EC_50_ of 6.5 and 6.1 nM (−8.07 and −8.05 log[M]), respectively (Fig. 4B). Gi and Go were also recruited at the same range of concentrations as β-Arrestins (Fig. 4C). Gq and G13 were not recruited (Fig. 4D).

When the concentration response curves are compared, it becomes clear that Gs activation occurs at a UCN2 concentration more than 10-fold less than the engagement of other proteins like Gi and β-Arrestin. The circulating levels of UCN2 observed in insulin resistant models (Fig. 1A, B) would likely engage predominantly Gs, whereas the levels of UCN2 following acute 1 mg/kg UCN2 dosing or in UCN2.AAV animals (Fig. 3I) would lead to significant β-Arrestin/Gi recruitment and potential desensitization.

β-Arrestin recruitment can sometimes cause the internalization of the receptor to which it is recruited. To assess whether CRHR2 is internalized following UCN2 treatment, we quantified CRHR2 receptor surface expression using confocal imaging of HEK cells transiently transfected with a plasmid expressing a C-terminus-HA-tagged CRHR2a construct (Fig. S3). Cells were treated with saline or 100 nM (−7.0 log[M]) UCN2 and surface expression was determined by fluorescent anti-HA antibody-binding, with and without permeabilizing the cell. The percentage of CRHR2 that is only visible when permeabilized is increased in cells treated with UCN2, indicating an increase of CRHR2 internalization and decreased surface receptor expression (Fig. 4E). Fluorescently labeled UCN2 (FAM-UCN2) is also internalized in a receptor-dependent manner (Fig. 4F). Live cell imaging shows that FAM-UCN2 accumulates at the cell surface of CRHR2-expressing cells in the first few hours of treatment, but then co-localizes with acidic compartments within the cell body over the next 24 h (Supplementary Movie 1 and 2). Cells that do not express CRHR2 neither recruit FAM-UCN2 to the cell surface nor internalize FAM-UCN2 (Supplementary Movie 3). CRHR2-expressing cells treated with saline also did not show recruitment or internalization of FAM signal (Supplementary Movie 4).

### Chronic UCN2 treatment reverses phosphorylation of CRHR2 targets in skeletal muscle

**Fig. 5:**
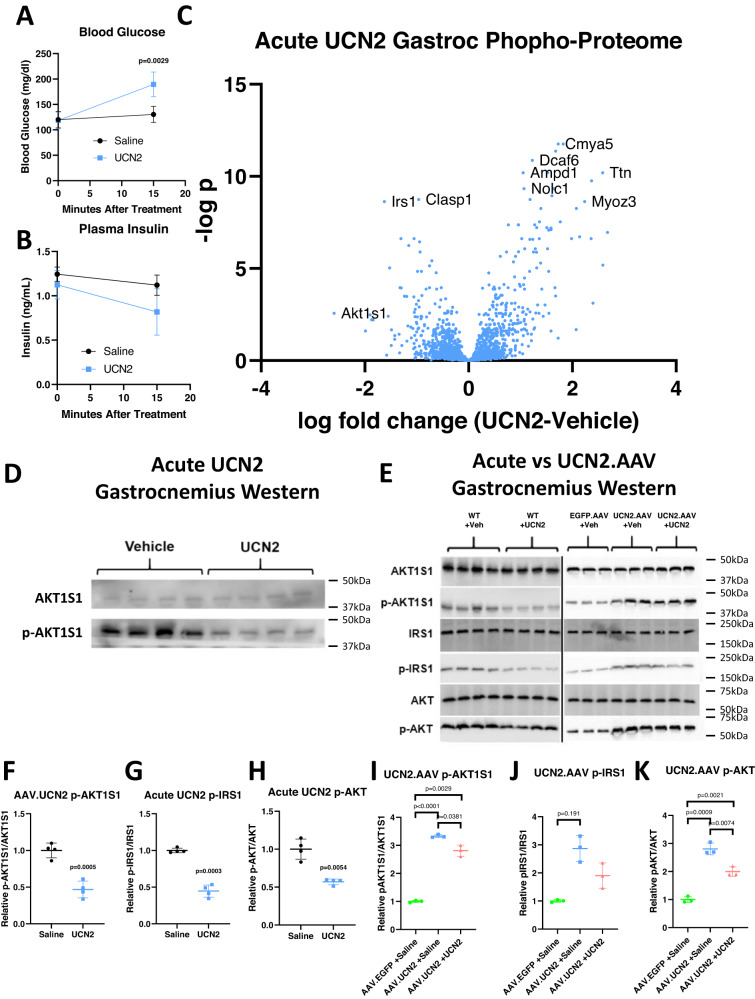
Chronic UCN2 treatment reverses UCN2 signaling in muscle. **A** Blood glucose levels 15 min following an intraperitoneal injection of saline or UCN2. *n* = 5 biologically independent animals. Significance was assessed by fitting longitudinal mixed effects models and one-way ANOVA. **B** Plasma insulin levels 15 min following an intraperitoneal injection of saline or UCN2. *n* = 5. Significance was assessed by fitting longitudinal mixed effects models and one-way ANOVA. **C** Phosphoproteomic volcano plot displaying relative differences in phosphorylation levels of protein in the gastrocnemius muscle from animals treated with saline or UCN2. *n* = 5. **D** Western blot of total AKT1S1 and p-AKT1S1 in gastrocnemius muscle from animals treated with saline or UCN2 for 15 min. Experiment was independently repeated once. **E** Western blot of total AKT1S1, total AKT1S1, total IRS1, p-AKT1S1, p-IRS1, and p-AKT in gastrocnemius muscle from animals treated with saline or UCN2 for 40 min and insulin for 20 min, or EGFP.AAV or UCN2.AAV for 7 days and insulin for 20 min. **F** Quantification of p-AKT1S1 western blot shown in (**E**) during acute saline of UCN2 treatment, normalized to total AKT1S1. *n* = 4 biologically independent animals. Significance was assessed by Welch’s two-way two sample *t*-test. **G** Quantification of p-IRS1 western blot shown in (**E**) during acute saline of UCN2 treatment, normalized to total IRS1. *n* = 4 biologically independent animals. Significance was assessed by Welch’s two-way two sample *t*-test. **H** Quantification of p-AKT western blot shown in (**E**) during acute saline of UCN2 treatment, normalized to total AKT. *n* = 4 biologically independent animals. Significance was assessed by Welch’s two-way two sample *t*-test. **I** Quantification of p-AKT1S1 western blot shown in (**E**) from EGFP.AAV or UCN2.AAV animals, normalized to total AKT1S1 *n* = 3 biologically independent animals. Significance was assessed by Welch’s two-way two sample *t*-test. **J** Quantification of p-IRS1 western blot shown in (**E**) from EGFP.AAV or UCN2.AAV animals, normalized to total IRS1 *n* = 3 biologically independent animals. Significance was assessed by Welch’s two-way two sample *t*-test. **K** Quantification of p-AKT western blot shown in (**E**) from EGFP.AAV or UCN2.AAV animals, normalized to total AKT *n* = 3 biologically independent animals. Significance was assessed by Welch’s two-way two sample *t*-test. All data are presented as mean values +/− SD. Source data are provided as a Source Data file.

To assess whether CRHR2 desensitization is occurring in vivo, we first set out to establish downstream targets of CRHR2 in metabolically active tissues. WT animals were injected with recombinant UCN2 and after 20 min, gastrocnemius muscle was removed and subjected to phosphoproteomic analysis. These UCN2-treated animals demonstrated significantly increased blood glucose levels and no significant difference in plasma insulin (Fig. 5A, B). The UCN2-treated gastrocnemius muscle phosphoproteome included >200 proteins that were significantly phosphorylated or dephosphorylated following UCN2 treatment (Figs. S4A, B and 5C). Pathway analysis was conducted on the phosphoproteome. The most significantly affected pathways included increased mTOR inhibitor pathways and decreased PDK phosphorylation pathways (Fig. S4C). Overall, the affected pathways were consistent with decreased nutrient uptake in the muscle.

Among the most dephosphorylated proteins were AKT1S1 and IRS1 (Fig. 5C). These proteins are of particular interest because of their involvement in insulin signaling. IRS1 is downstream of the insulin receptor and IGF1 receptor. It is phosphorylated in response to insulin binding, which activates PI3K and eventually leads to glucose uptake. AKT1S1 phosphorylation blocks the proteosomal degradation of IRS1, leading to increased glucose uptake. If these proteins are dephosphorylated following acute UCN2 treatment, that would translate into decreased PI3K activation, and decreased glucose uptake, which is consistent with the reduced glucose tolerance and uptake results following acute UCN2 treatment (Fig. 2A, H). The dephosphorylation of these proteins, along with AKT, with acute UCN2 treatment was confirmed by Western blot (Fig. 5D–H). Consistent with the desensitization hypothesis, animals chronically overexpressing UCN2 displayed the opposite change in signaling i.e., increased AKT1S1, IRS1, and AKT phosphorylation in skeletal muscle tissue (Fig. 5E, I–K).

### UCN2 pre-treatment desensitizes CRHR2 and promotes glucose uptake

**Fig. 6:**
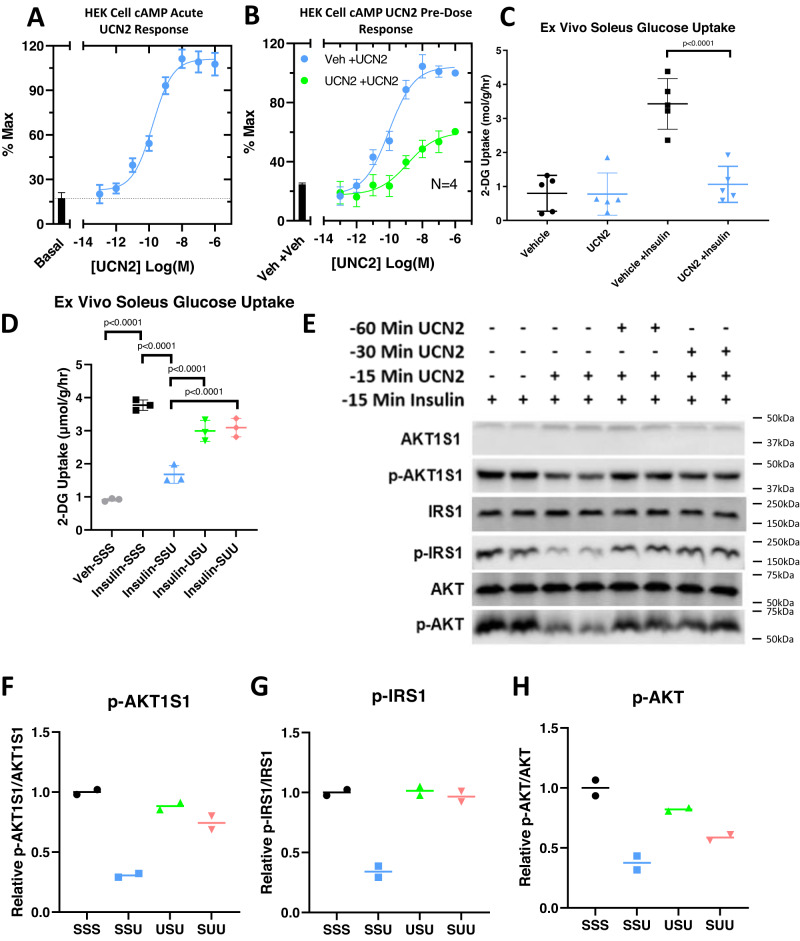
Repeated UCN2 treatment desensitizes CRHR2 and reverses effects of UCN2 directly on muscle. **A** Relative cAMP levels in transiently transfected HEK293 cells as a function of increasing doses of acute UCN2. *n* = 3 independent experiments. **B** Relative cAMP levels in transiently transfected HEK293 cells as a function of increasing doses of acute UCN2 following 6 h exposure to Saline or UCN2. *n* = 3 independent experiments. **C** [^3^H] 2-Deoxyglucose uptake in soleus muscle 20 min following treatment with saline, UCN2, saline +Insulin, or UCN2 +Insulin. *n* = 5 biologically independent animals. Significance was assessed by one-way ANOVA and Tukey HSD. **D** [^3^H] 2-Deoxyglucose uptake in mouse soleus muscle ex vivo. Muscles were treated with saline (SSS) or UCN2 at −15 min (SSU), at −15 and −30 min (SUU), or at −15 and −60 min (USU), +/− insulin at −15 min before glucose uptake was analyzed. *n* = 3 biologically independent animals. Significance was assessed by one-way ANOVA and Tukey HSD. **E** Western blot of total AKT1S1, total IRS1, total AKT1S1, p-AKT1S1, p-IRS1, and p-AKT in mouse soleus muscle ex vivo. Muscles were treated with aaline (SSS) or UCN2 at −15 min (SSU), at −15 and −30 min (SUU), or at −15 and −60 min (USU), +/− insulin at −15 min before being snap-frozen. Significance was assessed by one-way ANOVA and Tukey HSD. **F** Quantification of p-AKT1S1 western blot shown in (**E**), normalized to total AKT1S1 *n* = 2 biologically independent animals. **G** Quantification of p-IRS1 western blot shown in (**E**), normalized to total IRS1 *n* = 2 biologically independent animals. **H** Quantification of p-AKT western blot shown in (**E**), normalized to total AKT *n* = 2 biologically independent animals. All Data are presented as mean values +/− SD. Source data are provided as a Source Data file.

To determine whether CRHR2 functional desensitization in response to UCN2 occurred tissue-autonomously, we turned to CRHR2-transfected HEK 293 cells. Treating these cells with increasing concentrations of UCN2 produces a cAMP response that increases in a dose-dependent manner (Fig. 6A). Pre-treating these cells with UCN2, however, significantly blunts the cAMP activation response from subsequent UCN2 doses (Fig. 6B).

To determine whether UCN2 pre-exposure is sufficient to blunt the effects of UCN2 on glucose uptake within muscle, ex vivo studies were carried out on mouse soleus muscle. Soleus muscle was maintained at tension in muscle baths ex vivo. Acute treatment of the muscle with UCN2 was sufficient to blunt insulin-mediated glucose uptake (Fig. 6C). Pre-treating the muscle with UCN2 increased AKT1S1, IRS1, and AKT phosphorylation and increased insulin-mediated glucose uptake relative to acute UCN2 treatment (Fig. 6D–H), suggesting desensitization of CRHR2.

**Fig. 7:**
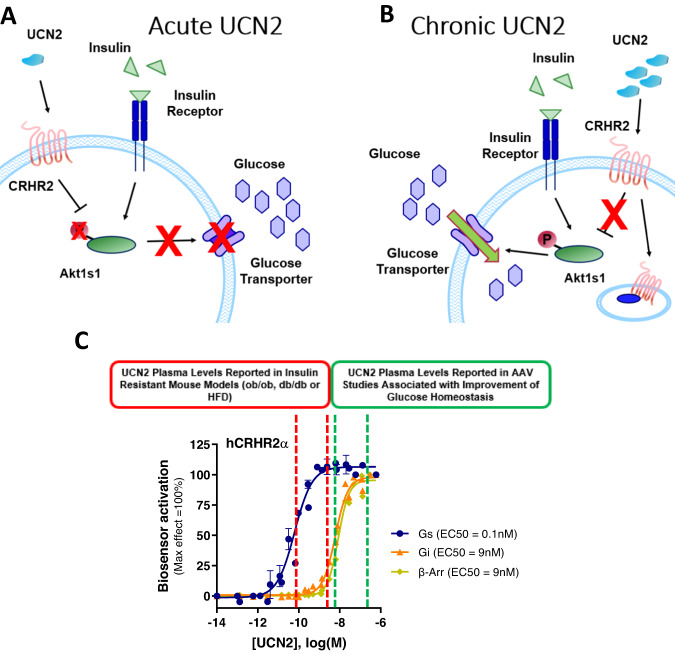
Schematic representation of acute and chronic effect of UCN2. **A** Acute UCN2 treatment impairs glucose uptake. **B** Chronic, high UCN2 treatment promotes glucose uptake. **C** Relative recruitment of Gs, Gi, bArrestin to CRHR2 with known circulating levels in various mouse models. All data are presented as mean values +/− SD.

The circulating levels of UCN2 reported in murine models of insulin resistance and in human patients with increased HOMA-IR fall in the range where we would expect to see mostly Gs recruitment to CRHR2 and very little alternative activation (Fig. 7C). The circulating levels reported in viral overexpression models would likely recruit significant levels of β-Arr, Gi, and Go to CRHR2 (Fig. 7C). Taken together, these results suggest that acute UCN2 acts directly on muscle cells through its binding with the GPCR CRHR2 to dephosphorylate AKT1S1 and decrease glucose uptake (Fig. 7A). On the other hand, sustained high levels of UCN2 can induce the desensitization of CRHR2 which will lead to increased p-AKT1S1 phosphorylation and increased glucose uptake by the skeletal muscle (Fig. 7B).

## Discussion

Obesity and associated metabolic disorders such as insulin resistance and Type 2 Diabetes (T2D) are major sources of morbidity and mortality that are reaching epidemic proportions^33–35^. Diet and exercise have proven to be effective in combating these afflictions; however, adherence to these interventions is generally low, and it is clear that additional treatments are needed to alleviate metabolic dysfunction^36–38^.

UCN2 and modified UCN2 peptides have been proposed as potential pharmaceutical treatments for insulin resistance in skeletal muscle^19,39,40^. However, the reported contradictory effects of UCN2 on whole body glucose metabolism and insulin sensitivity model organisms raises the question of what the effects of UCN2 treatment in patients would be, as well as the nature of signaling downstream of CRHR2. In this study we investigated the effects of acute and chronic UCN2 in mice. We found that acute dosing of recombinant UCN2 worsened glucose tolerance, while chronic overexpression of UCN2 improved it. This phenomenon appears to be carried out, at least in part, through direct effects on the skeletal muscle, as ex vivo experiments revealed that muscle incubated with UCN2 had reduced insulin-mediated glucose uptake, which was blunted by UCN2 pre-treatment.

UCN2 appears to have metabolically relevant effects on many tissues throughout the body. UCN2 regulates blood pressure in heart^41^, influences gastric motility^42^, and controls food intake centrally^12^. It is not surprising that a hormone seeking to influence systemic metabolism would have varied synergistic effects; however, it can complicate some conclusions. The animals overexpressing UCN2 in our study showed an initial reduction in food intake that was attenuated at day 7. How much this food intake reduction, and the resulting trends in fat mass, influenced the improvement in glucose tolerance is not known. However, similar trends were observed in the body weight and fat mass of animals treated with EGFP.AAV, and no difference was observed in glucose tolerance. The ex vivo experiments also indicate that at least some of this effect is carried out directly through skeletal muscle.

The model proposed in this report of CRHR2 desensitization potentially resolves the reported contradictions in UCN2’s effects on glucose uptake. Gs signaling has been shown to induce impaired glucose tolerance^43^, whereas Gi signaling improves insulin signaling^44,45^. In the current study, acute UCN2 dosing agonizes CRHR2 causing insulin resistance in the skeletal muscle, whereas chronic, elevated levels of UCN2 desensitize CRHR2 leading to the opposite: insulin sensitization. It appears that sustained Gi or β-Arrestin signaling is required to reverse the effects of acute UCN2 dosing.

These results mirror findings in studies on glucose-dependent insulinotropic polypeptide receptor (GIPR), another metabolically relevant GPCR. In these studies, a chronic high dose of long-acting GIPR agonist was shown to induce the internalization of GIPR^46^. The phenotypic outcomes of this treatment were similar to treatment with GIPR antagonists and GIP neutralizing antibody, that is to say, that both antagonism and chronic agonism led to decreased body weight and improved glucose uptake^47^.

The current study provides evidence that UCN2 can either improve skeletal muscle glucose uptake or impair it, depending on the dosing parameters. GPCRs are a major target group for pharmaceutical interventions. This work is important to understand the mechanisms involved in CRHR2 and GPCR signaling, in general. A drug that modulates skeletal muscle glucose uptake for the treatment of type 2 diabetes could have significant therapeutic impact, and these findings should be considered when determining the effectiveness of targeting CRHR2 or similar GPCRs for this purpose.

## Methods

### Mice

All animal experiments were conducted following study protocols and procedures reviewed and approved by Pfizer Institutional Animal Care and Use Committee. The facilities that supported this work are fully accredited by AAALAC International.Adult male wild-type (WT), ob/ob, lean ob+, db/db, and lean db+ mice (C57BL/6 J; 10−14 weeks old) were obtained from Jackson Laboratories (Farmington, CT). Mice were housed individually in Innovive cages (Innorack IVC Mouse 3.5) under a standard 12-h light/12-h dark cycle (06:00 h: 18:00 h) in a temperature- and humidity-controlled environment (22 ± 1 °C, 45 ± 2% humidity). Mice were given ad libitum access to tap water and standard chow (Innovive mouse metal feeder; Purina rodent diet 5061; Purina Mills, St. Louis, MO). Mice were used directly after importing into the animal facility following a minimum of 1week acclimation to housing conditions. Mice were also acclimated to handling and injection (0.1 mL saline) stress for ~5 days prior to the start of all studies. Body weight was recorded daily (Mettler Toledo ME4002TE, Mettler Toledo, Oakland, CA). Body composition was assessed on day 7 post-AAV treatment using the EchoMRI 4-in-1 500 Body Composition Analyzer (EchoMRI, Houston, TX). Mice were euthanized via carbon dioxide inhalation, followed by cervical dislocation and exsanguination by cardiac puncture. Data were assembled and visualized using Graphpad Prism (CA, USA). Data were assembled and visualized using Graphpad Prism (CA, USA).

### Pharmacokinetics

Plasma UCN2 and UCN3 measurements were made via ELISA according to manufacturer’s instructions (Kamiya Biomedical Company, Seattle, WA). Blood was collected via intracardiac stick. For ob/ob and db/db studies, blood was collected during ad lib feeding. For acute UCN2 dosing, blood was collected 20 min after dosing with UCN2 (Bachem, Bubendorf, Switzerland) dissolved in saline. For UCN2.AAV studies, unless otherwise noted, blood was collected on the seventh day after initial treatment.

### Quantitative RT-PCR

Human tissue cDNA was obtained from Zyagen (San Diego, CA). For mouse studies, animals were euthanized via CO2 and tissues were immediately dissected and snap frozen in liquid nitrogen. Ribonucleic acid (RNA) was extracted and purified using RNeasy RNA Isolation Kit (QIAGEN, Germantown, MD) then reverse transcribed into complementary deoxyribonucleic acid (cDNA) with a High-Capacity cDNA Reverse Transcription Kit (Applied Biosystems, Foster City, CA). Quantitative reverse transcriptase polymerase chain reaction (qRT-PCR) was performed using TaqMan reagents and primer-probes (Applied Biosystems, Foster City, CA). *Ucn2* (ThermoFisher, Mm01227928_s1) and *Ucn3* (ThermoFisher, Mm00453206_s1) genes were examined by qRT-PCR and normalized to the control gene TATA box binding protein (*Tbp*) (ThermoFisher, Mm01277042_m1).

### In vivo metabolic studies

All metabolic tests were performed using standard protocols on male littermates (aged: 10–14 weeks). For glucose tolerance tests (GTTs), animals were fasted for 8 h with ad libitum access to water. Fasting glucose measurements were taken before 2 mg/kg oral glucose dose. Blood glucose measurements were taken via tail nick using the AlphaTRAK 2 Glucose Monitoring System (Zoetis, Parsippany-Troy Hills, NJ) at 10, 30, 60, and 120 min after dose. Blood was collected for insulin measurement in EDTA tubes (Becton, Dickinson and Company, Franklin Lakes, NJ) and centrifuged to collect plasma (2000 g for 10 min at 4 °C), which was stored at −80 °C until analysis at 10, 30, 60, and 120 min after dose. Insulin levels were measured using an Ultrasensitive Mouse Insulin ELISA (ALPCO, Salem, NH), according to manufacturer’s instructions. For insulin tolerance tests (ITTs), animals were fasted for 8 h and blood glucose was measured before (0 min) and at 30, 60, 90, and 120 min following IP injection of 0.75 IU/kg of insulin (Humulin R, Eli Lilly, Indianapolis, IN). When indicated, mice were pre-injected IP with UCN2 (50–200 ug/kg, Bachem, Bubendorf, Switzerland).

### In vivo glucose uptake

Mice were fasted 5 h, dosed IP with 1 mg/kg UCN2 or vehicle. 20 min after dosing mice were injected with 2 mg/kg fluoro-deoxyglucose (FDG) +/− 0.75 IU insulin via tail vein. 15 min after the FDG dose animals were anesthetized and tissues and blood were collected and snap frozen. Plasma and tissues were analyzed for FDG, FDG-monophosphate, glucose, and glucose-monophosphate. The quantification of the analytes was performed by liquid chromatography tandem mass spectrometry (LC-MS/MS) using selected reaction monitoring (SRM) on an Applied Biosystems SCIEX (Framingham, MA, USA) 5500 QTrap mass spectrometer operated in negative ionization mode. Chromatography was achieved on a Shimadzu (Kyoto, Japan) HPLC system under reversed phase LC conditions with gradient elution. Calibration standards were prepared in analyte-free matrix, while quality control samples were prepared in pooled tissue homogenate by standard addition. Experimental tissue samples were mechanically homogenized in a methanol solution followed by evaporation under nitrogen and resuspension with injection solvent prior to analysis on the LC-MS/MS system.

To determine 2-deoxy-glucose (2DG) uptake in soleus and gastrocnemius muscle, mice were fasted for 5 h and anaesthetized with isoflurane. Animals were injected IP with 1 mg/kg UCN2 or a comparable volume of saline. 20 min later, the animals received a retroorbital injection of 0.4 uCi/g of body weight [^3^H]2DG (Perkin Elmer, Waltham, MA) and 0.75 IU/kg insulin in saline or a comparable volume of saline. 15 min following 2DG administration, blood was collected via intracardiac stick and soleus and gastrocnemius muscles were dissected and snap frozen in liquid nitrogen. Samples were stored at −80 C until processing. Blood was treated with BaOH2 and ZnSO4 and after centrifugation, radioactivity was determined via liquid scintillation counting (Tri-Carb 5110TR, PerkinElmer, Waltham, MA). Tissue was homogenized and mixed with water or BaOH2 and ZnSO4 to determine total and unphosphorylated 2DG content.

### Antisauvagine-30 studies

For glucose tolerance tests (GTTs), animals were fasted for 8 h with ad libitum access to water. Fasting glucose measurements were taken before 300ug/kg IP dose of ASG30 (Tocris Biosciences, Bristol, UK, Catalog No: 2071) or an equivalent volume of sterile distilled water. 10 min later animals received a 1 mg/kg IP dose of UCN2 or an equivalent volume of saline co-dosed with 300ug/kg of ASG30 or an equivalent volume of water. 20 min later animals received a 2 mg/kg oral glucose dose. Blood glucose measurements were taken via tail nick using the AlphaTRAK 2 Glucose Monitoring System (Zoetis, Parsippany-Troy Hills, NJ) at 10, 30, 60, and 120 min after dose.

To determine 2-deoxy-glucose (2DG) uptake in soleus muscle, mice were fasted for 5 h and anaesthetized with isoflurane. Animals were injected IP with 300 ug/kg of ASG30 or an equivalent volume of water. 10 min later the animals were dosed IP with 1 mg/kg UCN2 or a comparable volume of saline co-dosed with 300 ug/kg of ASG30 or an equivalent volume of water. 20 min later, the animals received a retroorbital injection of 0.4 uCi/g of body weight [^3^H]2DG (Perkin Elmer, Waltham, MA) and 0.75 IU/kg insulin in saline or a comparable volume of saline. 15 min following 2DG administration, blood was collected via intracardiac stick and soleus and gastrocnemius muscles were dissected and snap frozen in liquid nitrogen. Samples were stored at −80 C until processing. Blood was treated with BaOH2 and ZnSO4 and after centrifugation, radioactivity was determined via liquid scintillation counting (Tri-Carb 5110TR, PerkinElmer, Waltham, MA). Tissue was homogenized and mixed with water or BaOH2 and ZnSO4 to determine total and unphosphorylated 2DG content.

### Food intake studies

For acute studies, animals were singly housed and injected IP with saline or 1 mg/kg UCN2 (source). Food intake for the next 24 h was determined by measuring food weight in ceramic bowls within the cage. For the chronic studies, animals were singly housed and injected with Vehicle, EGFP.AAV, or UCN2.AAV. Food weight was measured in ceramic bowls every 24 h for 7 days.

### Viral dosing

AAV-EGFP and AAV-UCN2 were prepared at the Massachusetts General Hospital Vector Core Facility (Charlestown, MA). Vector construction and plasmid production was performed at LakePharma (Worcester, MA). All AAV’s were diluted in saline and administered at a dose of 2.0 × 10^10 ^g.c. as a single retroorbital injection (100 μl volume) under isoflurane anesthesia.

### Liver triglyceride content

On day 7 post-dose, EGFP.AAV and UCN2.AAV liver was dissected and snap-frozen in liquid nitrogen. 50–100 mg of pulverized tissue was homogenized and analyzed for triglyceride content using the Roche Hitachi 912 Analyzer Triglyceride Reagent Set (GMI, Ramsey, MN).

### CRHR2 BRET response curves

Effector membrane translocation biosensor assays (bioSensAll®) were conducted at Domain Therapeutics NA Inc. (Montreal, QC, Canada) and described in cells^28,29^. Assays were performed in HEK293 cells (Millipore Sigma), which were cultured in Dulbecco’s Modified Eagle Medium (DMEM) (Wisent # 319-015-CL) supplemented with 1% penicillin-streptomycin G (Wisent; cat# 450-201-EL) and 10% fetal bovine serum (Wisent # 090150) and maintained at 37 ^°^C with 5% CO2. All biosensor-coding plasmids and related information are the property of Domain Therapeutics. Transfections were performed using 25 kDa linear PEI (Polysciences, Warrington, PA) at a 3:1 ml of PEI/mg of DNA ratio. Briefly, DNA and PEI were diluted separately in 150 mM NaCl, mixed and then incubated for at least 20 min at room temperature (note: salmon sperm DNA (Invitrogen) was used to adjust the total amount of DNA transfected to a final quantity of 1 ug per mL of cell culture to be transfected). During the 20-minute incubation, HEK293 cells were detached, counted and resuspended into cell culture medium to a final density of 350,000 cells per mL. At the end of the 20-minute incubation, DNA/PEI complexes were added to cells followed by a gentle mixing. Cells were subsequently distributed in cell culture-treated 96-well plates (White Opaque 96-well Microplates, Greiner, cat# 655) at a density of 35,000 cells per well (i.e., 100 ul of cell suspension per well) and incubated at 37 ^°^C for 48 h. At 48 h post-transfection, the transfection medium was removed, and cells were washed once with 100 ul of Hank’s Balanced Salt Solution buffer (HBSS) (Wisent, cat#319-067CL: without red phenol; with sodium bicarbonate, with calcium and magnesium, with HEPES) per well. Wash buffer was then replaced by 100 ul of fresh HBSS per well and plates were incubated for 60 min at room temperature. At the end of this equilibration period, 10 ul of 10 mM e-Coelenterazine Prolume Purple (Methoxy e-CTZ; Nanolight, # 369) was added to each well followed immediately by the addition of increasing concentrations of Urocortin II using the HP D300 digital dispenser (Tecan). Cells were then incubated at room temperature for 10 min and BRET readings subsequently collected with a 0.4 s integration time on a Synergy NEO plate reader (BioTek Instruments, Inc., USA; filters: 400 nm/70 nm, 515 nm/20 nm). The BRET signal was calculated as the ratio of GFP10 emission to RlucII emission. All resulting dose response curves are represented as percent of max recruitment.

### HEK293 CRHR2 and FAM microscopy studies

HEK293 cells were plated at a density of 10,000 cells per well into tissue culture-treated clear bottomed, optical microscopy quality, 96 well plates (Perkin Elmer, PhenoPlate 96-well) and grown for 1 day to ~40–60% confluency. Twenty-four hours after plating, cells were transfected with 50 ng of pcDNA3.1(+) plasmid encoding a N-terminally tagged CRHR2α construct using Lipofectamine 3000 (Thermofisher) following manufacturing protocols. Twenty-four hours following transfection, agonists were then added for 30 min and cells were incubated at 37 °C. Prior to imaging, cells were washed once with PBS and fixed with ice-cold 4% paraformaldehyde in PBS for 15 min. After washing 3 times with PBS, cells were kept in the Alexa Fluor 647 anti-HA (1:2000 dilution, BioLegend cat# 682404) diluted in 5% normal goat serum in PBS. Two hours following antibody incubation at room temperature, the cells were washed three times in PBS and the nuclear stain Hoeschst 33342 (10ug/ml in PBS) and the acidic compartment stain, LysoTracker Deep Red (Thermofisher) was added for 30 min prior to imaging. A LSM880 Airyscan on a Carl Zeiss microscopy system was used for all image acquisition. Confocal laser excitation lines of 405 nm, 488 nm, and 633 nm were used for DAPI, Alexa fluor 488 (FAM), and Alexa fluor 647 fluorochromes, respectively using a Plan-Apochromat 63x/1.0 objective.

### cAMP assays

To measure cell-based CRHR2 agonist activity a HTRF (Homogenous Time-Resolved Fluorescence) cAMP detection kit (Cisbio cAMP dynamic 2 assay kit) was performed in non-induced HEK-TREX-CRHR2 alpha cells (Fisher Scientific). Cells were plated one day before the assay in 384-well plates at 1500 cells/well and assayed in adherent mode by removing the media, patting on paper towel and adding 10 µl of 1 µM of UCN2 or vehicle control for 1 h. Cells were gently washed 3 times with wash buffer and rested for 6 h in incubator. Following rest, cells were restimulated with titrating concentrations of UCN2 with IBMX, at a final concentration of 250 µM. Reactions continued for 30 min at 37 °C followed by addition of detection reagents and a one-hour minimum incubation at room temperature. HTRF, a competitive immunoassay, produces a fluorescence signal read with an Envision multi-plate reader using an excitation of 330 nm and emissions of 615 and 665 nm. By interpolation from a cAMP standard curve, raw data were converted to nm cAMP and then expressed as percent effect of 1 µM UCN2 in vehicle control pretreatment conditions.

### Phosphoproteomic tissue preparation

WT animals were injected IP with 1 mg/kg recombinant UCN2 or an equivalent volume of saline. Blood glucose and plasma insulin measurements were made pre-injection and 15 min post-dose. 20 min after the injection animals were euthanized and gastrocnemius muscle was dissected and snap-frozen in liquid nitrogen. The tissue was pulverized and homogenized in 700 ul SDS-buffer (0.1 M Tris/HCl 7.6, 4% SDS, 0.1 mM DTT), heated for 5 min at 95 °C and sonicated (Bioruptor®Plus Diagenode, 30 sec on-30sec off cycle for 10 cycles). After centrifugation for 10 min at 10,000 x g at 15 °C, the proteins in the supernatant were precipitated with 4X (volumes) ice-cold acetone (−20 °C) and stored at −20 °C overnight. The precipitates were centrifuged for 15 min at 10,000 × *g*, acetone was decanted, and the resulting pellets were dried for 10 min at room temperature. The pellet was resuspended in 500ul denaturing buffer (8 M urea and 10 mM Tris, pH 8.5) and sonicated (Bioruptor®Plus Diagenode, 30 s on-30 s off cycle for 5 cycles). The protein concentration was determined with a Bradford assay (Bio-Rad, Hercules, CA, USA). The proteins were reduced with dithiothreitol (DTT) 5 mM final concentration for 60 min at 37 °C and alkylated with iodoacetamide (8 mM final concentration) for 45 min at RT in the dark. Lys-C protease (Wako Chemicals, Richmond, VA, USA) was added at a ratio 1:100 to the total protein amount and incubated at 37 °C for 2 h. Urea was diluted to 2 M with 50 mM Tris pH8.5, and the samples were incubated with trypsin (1:50 w/w) (Pierce Biotechnology, USA) overnight at 37 °C at 600 rpm.

### Desalting and TMT labeling

The peptide mixtures were acidified with formic acid (FA) to 2% final concentration (pH < 3) and centrifuged at 4000 g for 5 min. The supernatants were transferred to reversed-phase C18 Sep-Pak cartridges (Waters, Milford, MA, USA) for desalting and concentration. Sep-Pak cartridges were prepared by sequential washing with 100% acetonitrile (ACN), 50% acetonitrile/0.1% FA and 0.1% Trifluoric acid (TFA) prior to loading of the peptide mixtures. Gravity was used for washing and loading of the samples. After sample loading, the Sep-Pak columns were washed with 0.1% TFA followed by 1% FA. Peptides were eluted from Sep-Pak with 50% ACN/0.1% FA. The eluted peptides were dried in a Speed-Vac centrifuge and reconstituted in 50 mM of HEPES to a final concentration of 1ug/ul, sonicated in water bath for 10 min. The peptide concentration was determined with a Pierce (#PI23275) peptide assay. 300ug of each sample was transferred to a fresh tube for TMT 10plex labeling (Thermo Scientific). The TMT kit was warmed to RT, and reconstituted in 41 ul of ACN, vortexed and incubated for 5 min.

The peptides were labeled according to the manufacturer’s instructions. To quench the reaction, 32 ul of 5% hydroxylamine was added and incubated for 15 min at RT at 1000 rpm. The labeled samples were combined and dried out in a Speed-Vac and peptides desalted using Sep-Pak cartridges (Waters, Milford, MA, USA).

### Off-line basic reversed-phase (High pH) high-pressure liquid chromatography (HPLC) fractionation

The samples were reconstituted in buffer A (4.5 mM ammonium formate (pH 10) in 2% (vol/vol) ACN) and sonicated in a water bath for 10 min. 90% of TMT labeled peptide mixture was fractionated using an Agilent 3.5-μm, 4.6 × 250 mm, model no. Zorbax 300 Extend-C18 column on an Agilent 1200 HPLC (Santa Clara, CA, USA) at 1 mL/min flow. Buffer A consisted of 4.5 mM ammonium formate (pH 10) in 2% (vol/vol) ACN and buffer B consisted of 4.5 mM ammonium formate (pH 10) in 90% (vol/vol) ACN; both buffers were adjusted to pH 10 with ammonium hydroxide. The gradient was programmed as following: 0–7 min 0% B, 7–13 min 16% B, 13–73 min 40% B, 73–77 min 44% B, 77–82 min 60% B, 82–96 min 60% B. Fractions were collected in 1 min interval in a 96 deep well plate. The fractions were pooled into 12 fractions (pooling all fractions that are 12 wells apart) plus the pooled flow through and dried in a Speed-Vac overnight.

### Phosphopeptide enrichment

Phosphopeptides from each pooled fraction were enriched on IMAC beads (Ni-NTA Superflow Agarose; Qiagen). For a 13-fraction IMAC enrichment, 160ul of beads (320ul of slurry) were washed 3X in 1 ml water and pelleted by centrifugation 1000 × *g* at RT for 1 min. The beads were resuspended in 1200 ul of 100 mM EDTA for 30 min at RT with end-over-end turning, and washed 3X in 1 ml water, 1000 × *g* at RT for 1 min. The beads were activated with 1200 ul of 10 mM iron (III) chloride aqueous solution (solid iron (III) chloride in HPLC water) for 30 min at RT with end-over-end turning. The beads were then washed 3X in 1 ml water, pelleted at 1000 × *g* at RT for 1 min, and resuspended in 460 ul of 1:1:1 (vol/vol/vol) ratio of ACN/MeOH/0.01% (vol/vol) Acetic Acid (AA). For each pooled fraction 40 ul of bead slurry (=10 ul of beads) were added to each of the 13 tubes. Each pooled fraction was resuspended in 190 ul of 50% (vol/vol) ACN/0.1% (vol/vol) TFA with vortexing. Once all the peptides were in solution, 285 ul of 100% (vol/vol) ACN/0.1% (vol/vol) TFA was added and then added to the aliquoted beads, incubated for 30 min at RT at 1000 rpm. After incubation the samples were spun down for 1 min at 1000 × *g* at RT and the supernatant was removed. 200 ul of 80% (vol/vol) ACN/0.1% (vol/vol) TFA were added to the beads.

### Elution and desalting of phosphopeptides

Peptides were desalted in 2-plug C18 (Empore C18 extraction disks) stage tips sequentially conditioned with 2 × 100% MeOH, 50% (vol/vol) ACN /0.1% (vol/vol) FA, and 2 × 1% (vol/vol) FA by centrifugation at 3000 g for 3 min. The sample on the beads was load onto the stage tip, centrifuged at 3000 g for 3 min at RT and washed sequentially with 2 × 80% (vol/vol) ACN/0.1% (vol/vol) TFA, 1% (vol/vol) FA, 500 mM K_2_PO_4_, and 1% (vol/vol) FA. The phospho-peptides were eluted with 50% (vol/vol) ACN in 0.1% (vol/vol) FA and dried by vacuum centrifugation.

### MS analyses

The peptides were loaded on a 50 cm column (Thermo Fisher ES903) and separated by reversed-phase chromatography using a gradient from 5 to 30% B over 2 hr (Buffer A: 0.1% FA in HPLC grade water; Buffer B: 80% ACN, 0.1% FA) with a flow rate of 0.25ul/min using an EASY-nLC 1200 system (Thermo Fisher Scientific).

For total proteome and phosphoproteome MS data were acquired on a Orbitrap Fusion Lumos mass spectrometer (Thermo Scientific) using a data-dependent acquisition top 10 method, AGC target 400000, maximum injection time of 50 msec, scan range of 300–1500 m/z and a resolution of 60 K. MS/MS was performed at a resolution of 50 K, AGC target 50000, maximum injection time of 86 msec, isolation window 0.7 m/z. Dynamic exclusion was set to 70 s.

Raw mass spectrometry data were processed using the MaxQuant software Version 1.6.7.0 (www.maxquant.org) with the Andromeda search engine integrated into MaxQuant environment. The MS/MS spectra were searched against the mouse UniProt sequence database without spliced isoforms. All MS/MS spectra were searched with the following MaxQuant parameters for peptide identification: acetyl (protein N-terminus) and methionine oxidation, were selected as variable modifications; cysteine carbamidomethylation was selected as fixed modification. For analyses of phosphopeptides, phosphorylation (STY) was added as variable modification. A maximum of 2 missed cleavages were allowed. Peptide spectrum matches and proteins were automatically filtered to a 1% false discovery rate based on Andromeda score, peptide length, and individual peptide mass errors. Modified peptides required a minimum peptide length of at least six amino acids (AA).

### Phosphoproteome quantification

TMT reporter ion intensity values were quantified from MS2 scans using an integration tolerance of 20 ppm with the most confident centroid setting (Maxquant 1.6.7.0) for matching peptides. Raw reporter ion abundance was used for further analysis. MSstatsTMT workflow starts from the peptide intensities reported in Maxquant’s evidence.txt file. When a peptide and charge combination was measured multiple times in a sample, only the maximum intensity was kept. The log2 peptide intensities were median normalized assuming equal input loading of all channels. Peptide intensities were summarized to protein intensities using Tukey’s median polish algorithm^48^.

Inspired on MSstatsTMT which builds protein-wise linear models, we developed in-house peptide-wise linear models based on peptide summaries using the same framework:

$${y}_{{st}}={\beta }_{0}+{\beta }_{{treatment}}+{\varepsilon }_{{st}}$$

${y}_{{st}}$ is the normalized log2-transformed peptide intensity in sample *s* of treatment *t*, ${\beta }_{0}$ is the intercept, ${\beta }_{{treatment}}$ is the effect of spike-in condition *t*, and, ${\varepsilon }_{{st}}$ is the protein-wise random error terms, which are assumed to be normally distributed with mean 0 and variance ${\sigma }^{2}$. The multiple testing problem is corrected using the Benjamini-Hochberg FDR procedure.

### Phosphosignature enrichment analysis

To identify phosphosignatures associated with UCN2 treatment, we applied the PTM-SEA algorithm to the differential analysis results from the UCN2 phosphoproteomics dataset^49^. The human PTMSigDB gene set was used for enrichment analysis, therefore the identified mouse phosphosites were mapped to their human orthologs as follows. The Mouse Genome Database was used to identify human orthologs, and the mouse and human protein sequences were aligned using the Needleman-Wunsch global alignment method^50^. The corresponding human site and +/− 7 flanking amino acids were extracted only. Sites falling in gaps during alignment were excluded. Next, signed log10 p-values were calculated from the UCN2/Saline differential analysis and used to rank the human ortholog phosphosites. PTM-SEA was performed using the ssGSEA2 available in the ssGSEA2.0 package (https://github.com/broadinstitute/ssGSEA2.0) with parameters sample.norm.type = “rank”, weight = 0, correl.type = “z.score”, statistic = “Kolmogorov-Smirnov”, min.overlap = 3, and nperm = 1000.

### Western blot

Animal tissues were snap-frozen in liquid nitrogen and stored at −80 °C. Frozen tissues were homogenized in ice-cold RIPA buffer (Sigma Aldrich, St. Louis, MO), and protein concentrations were determined using a BCA protein assay (Thermo Fisher Scientific, Waltham, MA). Protein extracts were separated on NuPAGE 4–20% Bis-Tris gels (Bio-Rad, Hercules, CA) and blotted onto PVDF membranes (Bio-Rad, Hercules, CA). Membranes were blocked for 1 h at room temperature in TBST (0.1%) containing 5% milk. Membranes were then incubated overnight at 4 °C with primary antibodies against pSer183-AKT1S1, total AKT1S1, pSer3-IRS1, total IRS1, pSer473-AKT, and total AKT (Cell Signaling, Danvers, MA #5936, #2610, #2385, #2382, #4060, and #4691, respectively) diluted 1:1000. Following three washing steps with TBST (0.1%), membranes were incubated with HRP-conjugated secondary antibodies for 1 h at room temperature. After thorough washing, proteins were visualized with SuperSignal West Dura Extended Duration Substrate (Thermo Fisher Scientific, Waltham, MA) on the Bio-Rad ChemiDoc MP. Immunoreactive bands were quantified using Image J Software (NIH).

### Ex vivo skeletal muscle glucose uptake and signaling assays

Soleus muscle was removed from fed animals anesthetized by an intraperitoneal injection of pentobarbital (10 mg/100 g body weight). Dissected soleus muscles were suspended at 5 mN of tension in incubation chambers (Model 610/820 M, Danish Myo-Technology, Denmark) containing Krebs-Ringer-Buffer supplemented with 0.1% BSA, 2 mM pyruvate and 8 mM Mannitol. During the entire incubation period the KRB was maintained at 30 °C and oxygenated with 95% O_2_ and 5% CO_2_. Following 10 min of pre-incubation muscles were incubated in Krebs buffer +/− 100 nM (−7.0 log[M]) UCN2. At the next time point, 30 min later, muscles were placed in baths containing Krebs buffer +/− 100 nM (−7.0 [M]) UCN2. At the next time point, 15 min later, muscles were placed in baths containing Krebs buffer +/− 100 nM (−7.0 log[M]) UCN2 + /− 60 nM Insulin (Actrapid, Novo Nordisk, Bagsvaerd, Denmark) for 15 min. For the ex vivo glucose uptake studies, 1 mM [^3^H]2-deoxyglucose (Perkin-Elmer) and 7 mM [^14^C]mannitol (Perkin-Elmer) was also added at this time. Muscles that were in saline in the first bath, saline in the second bath, and saline in the third bath were assigned to the SSS group. Muscles that were in saline in the first bath, saline in the second bath, and UCN2 in the third bath were assigned to the SSU group. Muscles that were in UCN2 in the first bath, saline in the second bath, and UCN2 in the third bath were assigned to the USU group. Muscles that were in saline in the first bath, UCN2 in the second bath, and UCN2 in the third bath were assigned to the SUU group. Following incubation, muscles were harvested, washed in ice-cold KRB and immediately frozen in liquid nitrogen.

Glucose uptake was determined in muscle protein lysates by assessing the accumulation of [^3^H]2-deoxyglucose with the use of [^14^C]mannitol to correct for the extracellular space. Radioactivity was measured by liquid scintillation counting.

For insulin-signaling, muscles were pulverized and homogenized in RIPA buffer. Protein samples were run in SDS-PAGE gels and blotted with antibodies against AKT1S1, IRS1, AKT, p-AKT1S1, p-IRS1, and p-AKT.

### Statistics

Statistical parameters including the exact sample size (n), post-hoc tests, and statistical significance are reported in figure legends. Data are expressed as mean +/− standard deviation. Data were estimated to be statistically significant when *P* < 0.05.

### Reporting summary

Further information on research design is available in the Nature Portfolio Reporting Summary linked to this article.

## Supplementary information

Supplementary Information

Description of Additional Supplementary Files

Supplementary Movie 1

Supplementary Movie 2

Supplementary Movie 3

Supplementary Movie 4

Reporting Summary

## Data Availability

All data are available in the main text of Supplementary Materials. The phosphoproteomics data generated in this study have been deposited to the deposited to the MassIVE repository (https://massive.ucsd.edu/ProteoSAFe/dataset.jsp?accession=MSV000092094). Source data are provided with this paper.

## Source data

Source Data
