# Supplementary material for: Chronic UCN2 treatment desensitizes CRHR2 and improves insulin sensitivity
Source: Nat Commun. 2023 Jul 4;14:3953. doi: 10.1038/s41467-023-39597-w (PMC10319809; doi:10.1038/s41467-023-39597-w)
Supplement: Supplementary file 1 — Supplementary Information [file 41467_2023_39597_MOESM1_ESM.pdf]

Supplementary Fig. 1

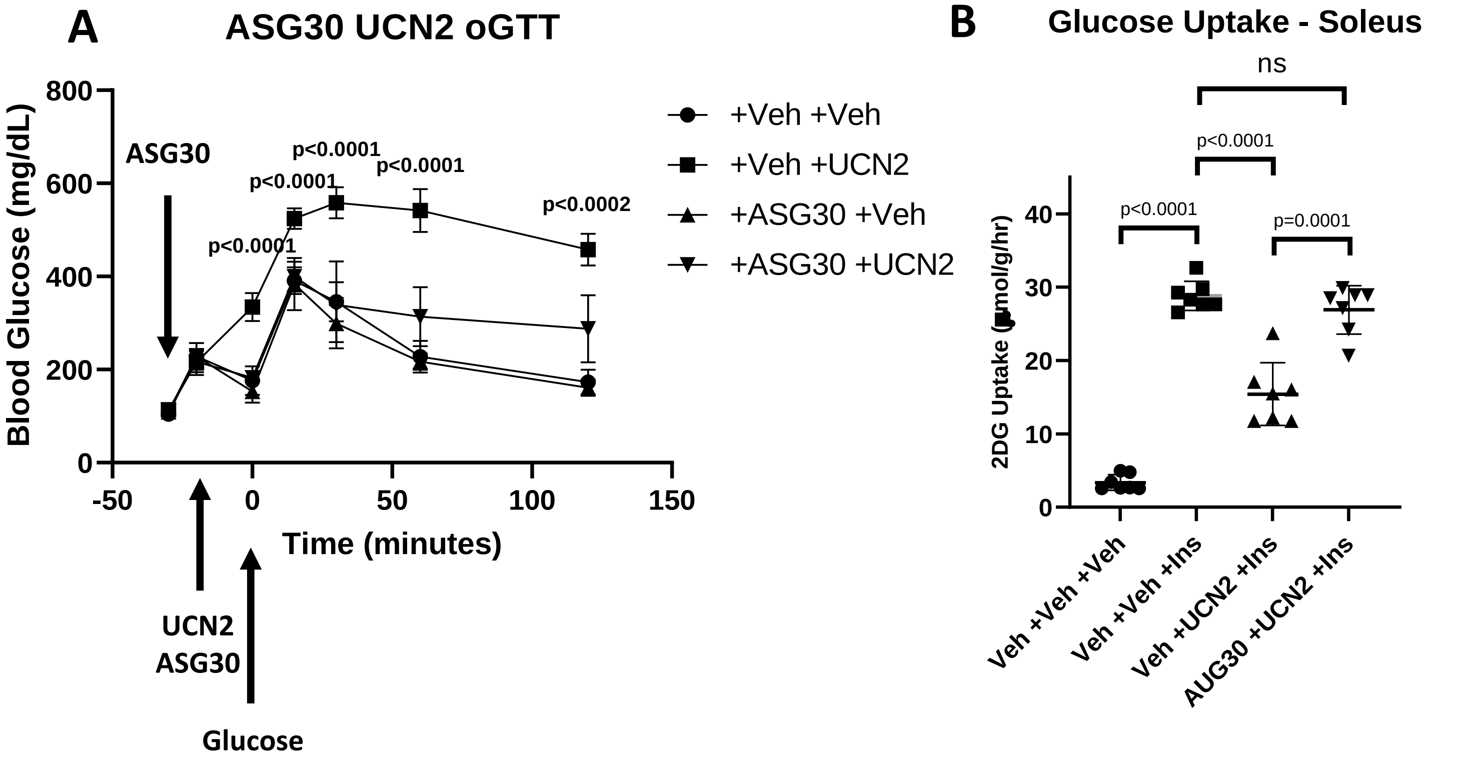

Supplementary Fig. 1. Antisauvagine-30 Blunts Effects of UCN2 on Glucose Uptake.

(A) Blood glucose during oGTT. Oral glucose gavage occurred at t = 0. At t = -30 animals were treated with either Antisauvagine-30 (ASG30) or water. At t = -20 animals were treated with either Saline, UCN2 alone, ASG30 alone, or ASG30 and UCN2 together. Mean±SD, n = 6. Significance was assessed by one-way ANOVA and Tukey HSD.

(B) [^{3}H]2-Deoxyglucose uptake by the soleus muscle in insulin-treated or untreated mice, following pre-treatment of saline, UCN2, or ASG30 and UCN2. Mean±SD, n = 6.

Significance was assessed by one-way ANOVA and Tukey HSD. * P < 0.05, ** P < 0.01, *** P < 0.001, **** P < 0.0001.

Supplementary Fig. 2

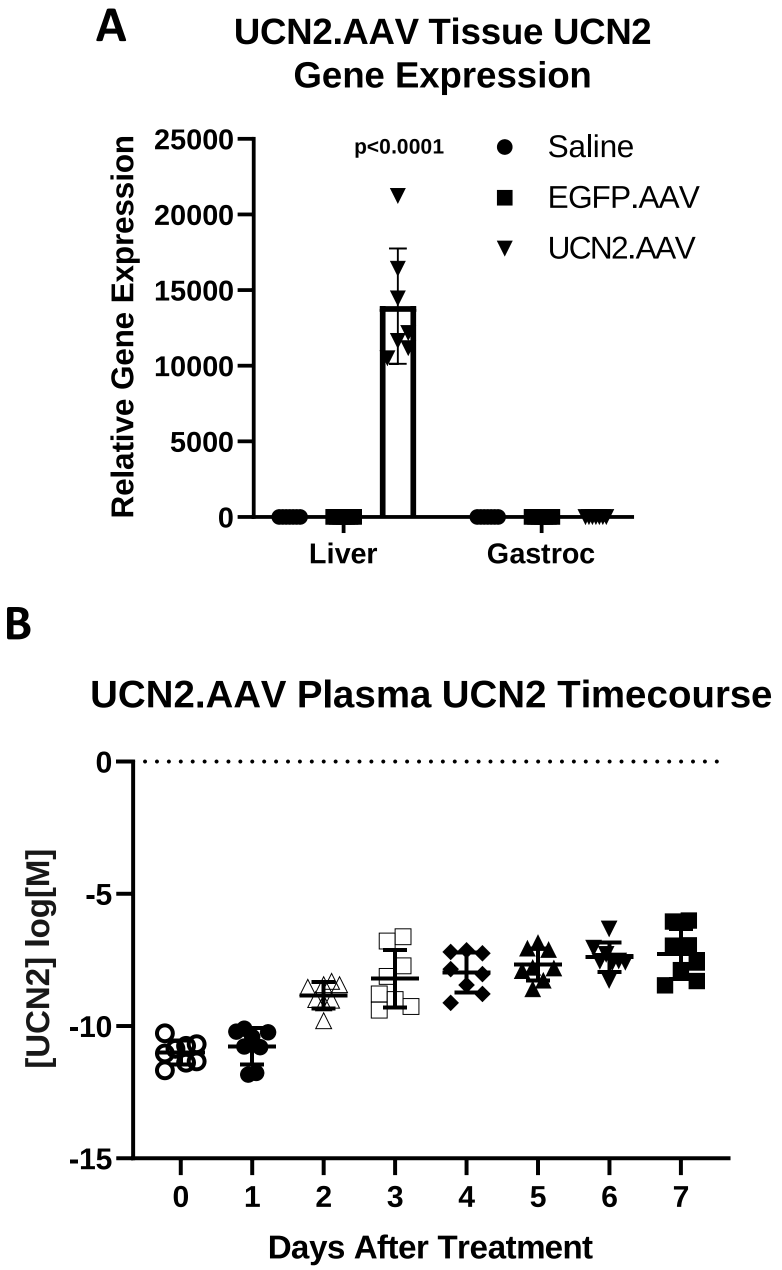

Supplementary Fig. 2. UCN2.AAV Dosing.

A. U*cn2* gene expression in liver and gastrocnemius muscle from saline-dosed, EGFP.AAV-dosed, and UCN2.AAV-dosed animals, 7 days after treatment. Mean±SD, n = 8. Significance was assessed by one-way ANOVA and Tukey HSD.
B. Serum UCN2 concentrations taken each day after treatment with UCN2.AAV. Mean±SD, n = 8.

* P < 0.05, ** P < 0.01, *** P < 0.001, **** P < 0.0001 .

Supplementary Figure 3

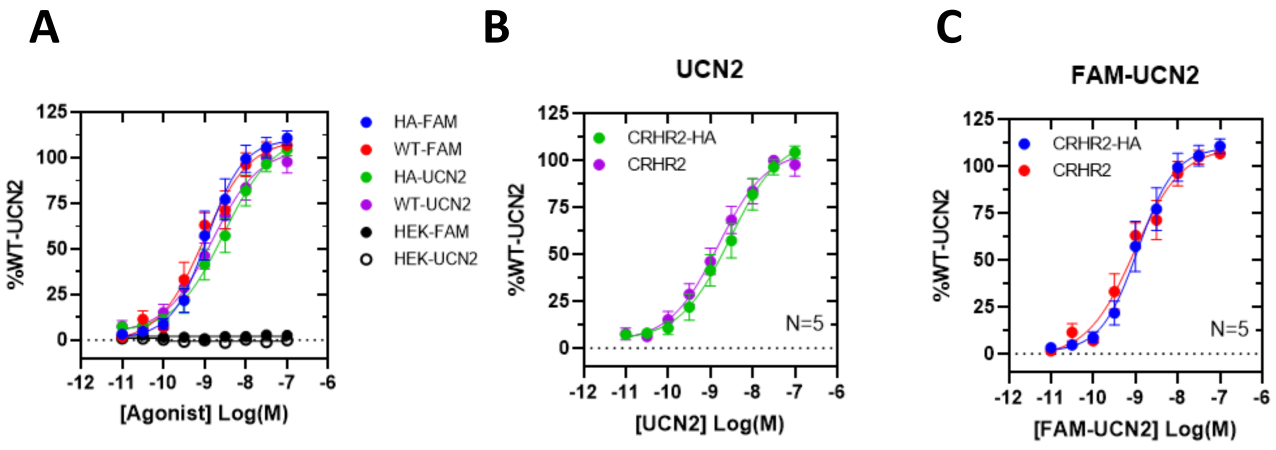

D

	HA-FAM			WT-FAM			HA-UCN			WT-UCN		
	Mean	SEM	N	Mean	SEM	N	Mean	SEM	N	Mean	SEM	N
Bottom	4.3e-004	3.6	7	1.1	3.4	7	4.8	2.2	5	3.8	3.7	5
Top	112.8	4.2	7	112.7	1.9	7	115.3	4.6	5	109.0	4.5	5
LogEC50	-9.1	0.2	7	-9.1	0.2	7	-8.5	0.2	5	-8.8	0.2	5
HillSlope	1.4	0.2	7	1.0	0.2	7	0.8	3.3e-002	5	0.8	0.1	5
EC50	1.4e-009	5.0e-010	7	1.2e-009	4.2e-010	7	4.6e-009	2.0e-009	5	2.2e-009	8.4e-010	5
Span	112.7	6.5	7	111.6	4.3	7	110.6	5.3	5	105.1	7.4	5

Supplementary Fig. 3. UCN2 Dose-Response Time Course.

(A) Intensity of CRHR2 downstream signaling in response to increasing doses of recombinant human UCN2 or FAM-labeled UCN2 in reporter HEK293 cells transfected with human CRHR2. n = 1.

(B) Intensity of CRHR2 downstream signaling in response to increasing doses of recombinant human UCN2 in reporter HEK293 cells transfected with WT human CRHR2 or HA-tagged CRHR2. n = 1.

(C) Intensity of CRHR2 downstream signaling in response to increasing doses of FAM-labelled UCN2 in reporter HEK293 cells transfected with WT human CRHR2 or HA-tagged CRHR2. n = 1 .

(D) Dose-response curve characteristics in HEK293 cells with WT human CRHR2 (WT) or HA-tagged human CRHR2 following treatment with human recombinant UCN2 or FAM-labeled UCN2.

Supplementary Fig. 4

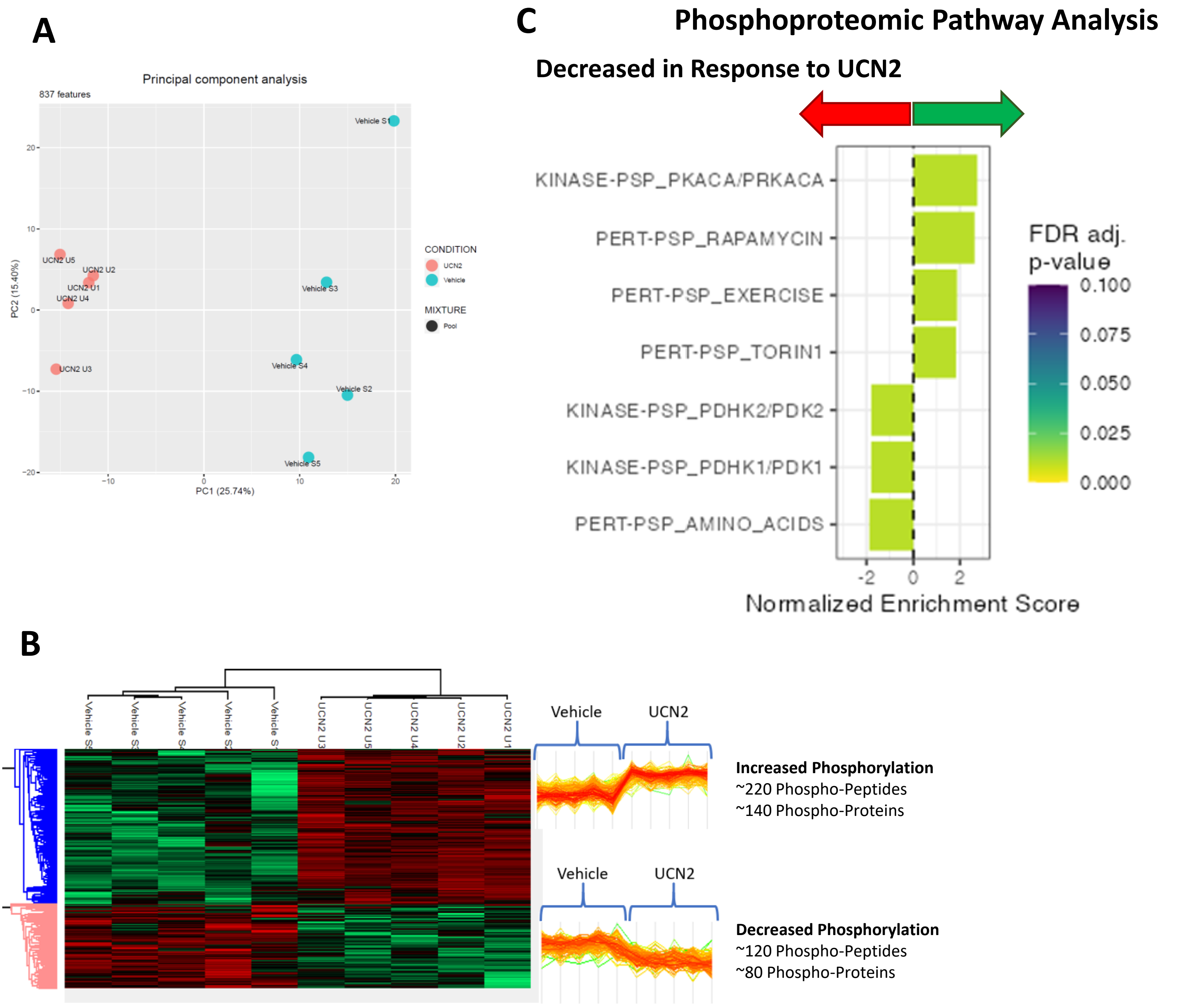

Supplementary Fig. 4. UCN2 Gastrocnemius Muscle Phosphoproteomics.

(A) Principal component analysis of the phosphoproteomes of saline and UCN2-treated gastrocnemius muscle. n = 5.

(B) Heatmap of proteins phosphorylated or dephosphorylated in response to UCN2 treatment. n = 5.

(C) Phosphoproteomic pathway analysis for saline and UCN2-treated gastrocnemius muscle. n = 5.
