# Supplementary material for: Chronic UCN2 treatment desensitizes CRHR2 and improves insulin sensitivity
Source: Nat Commun. 2023 Jul 4;14:3953. doi: 10.1038/s41467-023-39597-w (PMC10319809; doi:10.1038/s41467-023-39597-w)
Supplement: Supplementary file 2 — Description of Additional Supplementary Files [file 41467_2023_39597_MOESM2_ESM.pdf]

Description of Additional Supplementary Files

Supplementary Movie 1

Confocal microscopy of live HEK293 cells, transfected with human CRHR2 and treated with FAM-UCN2 over 24 hours. Blue = Hoescht (nuclei), Red = Lysostracker (acidic compartments), and Green = FAM-UCN2. Scale bars = 10μm.

Supplementary Movie 2

Confocal microscopy of live HEK293 cells, transfected with human CRHR2 and treated with FAM-UCN2 over 24 hours. Blue = Hoescht (nuclei), Red = Lysostracker (acidic compartments), and Green = FAM-UCN2. Scale bars = 10μm.

Supplementary Movie 3

Confocal microscopy of live WT HEK293 cells treated with FAM-UCN2 over 24 hours. Blue = Hoescht (nuclei), Red = Lysostracker (acidic compartments), and Green = FAM-UCN2. Scale bars = 10μm.

Supplementary Movie 4

Confocal microscopy of live HEK293 cells, transfected with human CRHR2 and treated with saline over 24 hours. Blue = Hoescht (nuclei), Red = Lysostracker (acidic compartments), and Green = FAM-UCN2. Scale bars = 10μm.
