# Supplementary material for: Chronic UCN2 treatment desensitizes CRHR2 and improves insulin sensitivity
Source: Nat Commun. 2023 Jul 4;14:3953. doi: 10.1038/s41467-023-39597-w (PMC10319809; doi:10.1038/s41467-023-39597-w)
Supplement: Supplementary file 7 — Reporting Summary [file 41467_2023_39597_MOESM7_ESM.pdf]

nature portfolio

Corresponding author(s): Zhidan Wu

Last updated by author(s): Jun 5, 2023

Reporting Summary

Nature Portfolio wishes to improve the reproducibility of the work that we publish. This form provides structure for consistency and transparency in reporting. For further information on Nature Portfolio policies, see our Editorial Policies and the Editorial Policy Checklist.

Statistics

For all statistical analyses, confirm that the following items are present in the figure legend, table legend, main text, or Methods section.

n/a	Confirmed	
☐	☒	The exact sample size (*n*) for each experimental group/condition, given as a discrete number and unit of measurement
☐	☒	A statement on whether measurements were taken from distinct samples or whether the same sample was measured repeatedly
☐	☒	The statistical test(s) used AND whether they are one- or two-sided<br>*Only common tests should be described solely by name; describe more complex techniques in the Methods section.*
☐	☒	A description of all covariates tested
☐	☒	A description of any assumptions or corrections, such as tests of normality and adjustment for multiple comparisons
☐	☒	A full description of the statistical parameters including central tendency (e.g. means) or other basic estimates (e.g. regression coefficient) AND variation (e.g. standard deviation) or associated estimates of uncertainty (e.g. confidence intervals)
☐	☒	For null hypothesis testing, the test statistic (e.g. *F*, *t*, *r*) with confidence intervals, effect sizes, degrees of freedom and *P* value noted<br>*Give P values as exact values whenever suitable.*
☒	☐	For Bayesian analysis, information on the choice of priors and Markov chain Monte Carlo settings
☒	☐	For hierarchical and complex designs, identification of the appropriate level for tests and full reporting of outcomes
☒	☐	Estimates of effect sizes (e.g. Cohen's *d*, Pearson's *r*), indicating how they were calculated

Our web collection on statistics for biologists contains articles on many of the points above.

Software and code

Policy information about availability of computer code

Data collection	Microsoft Excel v2208 (WA, USA), Graphpad Prism 9 (CA, USA)
Data analysis	Microsoft Excel v2208 (WA, USA), Graphpad Prism 9 (CA, USA), ImageJ (https://imagej.nih.gov), MaxQuant v1.6.7.0 (Munich, Germany)

For manuscripts utilizing custom algorithms or software that are central to the research but not yet described in published literature, software must be made available to editors and reviewers. We strongly encourage code deposition in a community repository (e.g. GitHub). See the Nature Portfolio guidelines for submitting code & software for further information.

Data

Policy information about availability of data

All manuscripts must include a data availability statement. This statement should provide the following information, where applicable:

- Accession codes, unique identifiers, or web links for publicly available datasets
- A description of any restrictions on data availability
- For clinical datasets or third party data, please ensure that the statement adheres to our policy

All data are available in the main text of Supplementary Materials. Source data are provided with this paper. The phosphoproteomics data generated in this study have been deposited to the deposited to the MassIVE repository (https://massive.ucsd.edu/) with the dataset identifier MSV000092094.

Research involving human participants, their data, or biological material

Policy information about studies with human participants or human data. See also policy information about sex, gender (identity/presentation), and sexual orientation and race, ethnicity and racism.

Reporting on sex and gender	Not applicable
Reporting on race, ethnicity, or other socially relevant groupings	Not applicable
Population characteristics	Not applicable
Recruitment	Not applicable
Ethics oversight	Not applicable

Note that full information on the approval of the study protocol must also be provided in the manuscript.

Field-specific reporting

Please select the one below that is the best fit for your research. If you are not sure, read the appropriate sections before making your selection.

[x] Life sciences [ ] Behavioural & social sciences [ ] Ecological, evolutionary & environmental sciences

For a reference copy of the document with all sections, see nature.com/documents/nr-reporting-summary-flat.pdf

Life sciences study design

All studies must disclose on these points even when the disclosure is negative.

Sample size	Sample size was determined using power analysis, taking into account effect size based on pilot experiments.
Data exclusions	No data were excluded.
Replication	Ob/ob, db/db, DIO, experiments were run once.<br>Human tissue studies were run once.<br>Acute UCN2 treatment studies were run twice.<br>Chronic UCN2.AAV treatment studies were run twice.<br>G-protein recruitment BRET studies were run two-three times (Gs three times, Gi three times, Go twice, bArr1 three times, bArr2 twice, Gq twice, G13 twice).<br>HEK CRHR2 internalization studies were run four times.<br>Phosphoproteomic analysis studies were run once.<br>Confirmatory western blots were run twice.<br>HEK cell cAMP response studies were run four times.<br>Ex vivo soleus glucose uptake studies were run twice.
Randomization	For both acute and chronic in vivo treatment, animals were randomized by body weight prior to the start of the studies. Ex vivo studies were randomized by donor animal body weight. Other experiments are not subjective but rather based on results from quantitative analysis software.
Blinding	All in vivo and ex vivo experiments were carried out by researchers that were blinded to treatment group. Other experiments are not subjective but rather based on results from quantitative analysis software.

Reporting for specific materials, systems and methods

We require information from authors about some types of materials, experimental systems and methods used in many studies. Here, indicate whether each material, system or method listed is relevant to your study. If you are not sure if a list item applies to your research, read the appropriate section before selecting a response.

Materials & experimental systems

n/a	Involved in the study
☐	☒ Antibodies
☐	☒ Eukaryotic cell lines
☒	☐ Palaeontology and archaeology
☐	☒ Animals and other organisms
☒	☐ Clinical data
☒	☐ Dual use research of concern
☒	☐ Plants

Methods

n/a	Involved in the study
☒	☐ ChIP-seq
☒	☐ Flow cytometry
☒	☐ MRI-based neuroimaging

Antibodies

Antibodies used	Alex Fluor 647 anti-HA (Alexa 647-conjugated, clone: 16B12, BioLegend, cat# 682404, dilution 1:1000)<br>pSer183-AKT1S1 (unconjugated, clone: unspecified, Cell Signaling, cat# 5936, dilution 1:1000)<br>total AKT1S1 (unconjugated, clone: unspecified, Cell Signaling, cat# 2610, dilution 1:1000)<br>pSer3-IRS1 (unconjugated, clone: unspecified, Cell Signaling, cat# 2385, dilution 1:1000)<br>total IRS1 (unconjugated, clone: unspecified, Cell Signaling, cat# 2382, dilution 1:1000)<br>pSer473-AKT (unconjugated, clone: unspecified, Cell Signaling, cat# 4060, dilution 1:1000)<br>total AKT (unconjugated, clone: unspecified, Cell Signaling, cat# 4691, dilution 1:1000)<br>Anti-rabbit IgG, HRP-linked Antibody (HRP-conjugated, clone: unspecified, Cell Signaling, cat# 7074, dilution 1:1000)
Validation	Alex Fluor 647 anti-HA (BioLegend, cat# 682404) RRID:AB_25<br>pSer183-AKT1S1 (Cell Signaling, cat# 5936) RRID:AB_10838139<br>total AKT1S1 (Cell Signaling, cat# 2610) RRID:AB_916206<br>pSer3-IRS1 (Cell Signaling, cat# 2385) RRID:AB_330363<br>total IRS1 (Cell Signaling, cat# 2382) RRID:AB_330333<br>pSer473-AKT (Cell Signaling, cat# 4060) RRID:AB_2315049<br>total AKT (Cell Signaling, cat# 4691) RRID:AB_915783<br>Anti-rabbit IgG, HRP-linked Antibody (Cell Signaling, cat# 7074) RRID:AB_2099233

Eukaryotic cell lines

Policy information about cell lines and Sex and Gender in Research

Cell line source(s)	HEK293 (Millipore Sigma), Hlp-In T-Rex HEK293 (ThermoFisher Scientific)
Authentication	Cell lines were authenticated using PCR assays with species-specific primers
Mycoplasma contamination	Cell lines tested negative for mycoplasma contamination
Commonly misidentified lines<br>(See ICLAC register)	No commonly misidentified lines were used in this study

Animals and other research organisms

Policy information about studies involving animals; ARRIVE guidelines recommended for reporting animal research, and Sex and Gender in Research

Laboratory animals	Studies were conducted using adult male wild-type (WT), ob/ob, lean ob+, db/db, and lean db+ mice (C57BL/6J; 10-14 weeks old) obtained from Jackson Laboratories (Farmington, CT). Mice were housed individually in Innovive cages (Innorack IVC Mouse 3.5) under a standard 12-h light/12-h dark cycle (06:00 h: 18:00 h) in a temperature- and humidity-controlled environment (22 ± 1°C, 45 ± 2% humidity). Mice were given ad libitum access to tap water and standard chow (Innovive mouse metal feeder; Purina rodent diet 5061; Purina Mills, St. Louis, MO). Mice were used directly after importing into the animal facility following a minimum of 1 week acclimation to housing conditions. Mice were also acclimated to handling and injection (0.1 mL saline) stress for ~5 days prior to the start of all studies.
Wild animals	The study did not involve wild animals
Reporting on sex	The reported studies were carried out in male animals. Many studies were also carried out in female animals with similar results.
Field-collected samples	This study did not involve field-collected samples
Ethics oversight	All animal experiments were conducted following study protocols reviewed and approved by Pfizer Institutional Animal Care and Use Committee. The facilities that supported this work are fully accredited by AAALAC International.

Note that full information on the approval of the study protocol must also be provided in the manuscript.
